# Nicotinamide riboside reduces glial inflammation and boosts mitochondrial function

**DOI:** 10.7150/ijbs.119262

**Published:** 2026-06-04

**Authors:** Tsering Yangzom, Anbin Chen, Bjørn Christian Lundberg, Kristina Xiao Liang

**Affiliations:** 1 Department of Biomedicine (IBM), University of Bergen, Bergen, Norway.; 2 Department of Neurosurgery, Xinhua Hospital Affiliated with Shanghai Jiaotong University School of Medicine, Shanghai, China.; 3 Department of Clinical Medicine (K1), University of Bergen, Bergen, Norway.

## Abstract

Astrocyte dysfunction plays a pivotal role in the pathogenesis of POLG-related mitochondrial diseases, yet the underlying mechanisms remain poorly understood. Here, we employed human iPSC-derived astrocytes, cortical organoids and astrocyte-neuron co-culture systems to model *POLG* mutations and investigate astrocyte-mediated neurotoxicity. Single-cell transcriptomic profiling revealed a marked expansion of A1 neurotoxic astrocytes, depletion of A2 neuroprotective astrocytes, and reduction of neuronal populations in POLG organoids. A1 astrocytes exhibited transcriptional signatures of mitochondrial dysfunction, inflammatory signaling (TGF-β, JAK-STAT), impaired neuro-supportive functions, and activation of senescence, autophagy, and proteostasis stress pathways. Co-cultured dopaminergic neurons displayed impaired morphology and widespread transcriptional downregulation of mitotic, cytoskeletal, and synaptic genes, along with activation of inflammatory and ion transport pathways. Treatment with the NAD⁺ precursor nicotinamide riboside (NR) attenuated astrocyte reactivity, reduced IL-6 and CXCL1 secretion, improved neuronal structure and synaptic marker expression, and increased mtDNA copy number and ATP production in POLG astrocytes. Our study identifies NAD⁺ augmentation as a promising strategy to mitigate astrocyte-driven pathology in mitochondrial encephalopathies.

## Introduction

Mitochondria are essential organelles that regulate energy metabolism, redox balance, calcium signaling, and cell survival. These functions are especially critical in the central nervous system (CNS), where neurons depend heavily on mitochondrial ATP production and homeostasis. Defective mitochondrial function is increasingly recognized as a central feature^1^,^2^ of a broad range of neurodegenerative diseases^3^-^5^ such as Parkinson's and Alzheimer's, impacting around 10% of the population^6^,^7^, and also contributes to diabetic retinopathy, the leading cause of blindness in adults. Despite extensive studies in neurons, the contribution of non-neuronal cells-especially astrocytes-to mitochondrial dysfunction and neurodegeneration is still underappreciated.

Among mitochondrial disorders, mutations in the *POLG* gene, which encodes polymerase gamma (pol γ)^8^ involved in mitochondrial DNA (mtDNA) replication, is linked to various mitochondrial disorders through mutations that cause mitochondrial dysfunction and mtDNA maintenance issues. These mutations result in tissue-specific mtDNA aberrations, significantly affecting neurons and leading to neuronal loss due to mtDNA depletion and deletion^9^, resulting in mitochondrial respiratory chain defects, impaired oxidative phosphorylation, and progressive neurological decline. Clinically, POLG-related disorders present with epilepsy, cognitive dysfunction, motor impairments, and neurodegenerative-symptoms that point toward dysfunction not only in neurons but also in the broader neuroglial environment.

Emerging evidence suggests that astrocytes, the most abundant glial cell type in the CNS, play a central role in the pathogenesis of various neurodegenerative diseases^10^-^14^. Beyond their traditional supportive functions, astrocytes are essential for maintaining neuronal health by regulating synaptic transmission, providing metabolic and trophic support^15^,^16^, and contributing to neurovascular coupling^17^. Despite their critical functions, the role of astrocytes in mitochondrial diseases remains poorly understood. Under pathological conditions, astrocytes can shift into reactive states that may be either neuroprotective or neurotoxic, depending on the context^12^. These reactive phenotypes influence the progression of neurodegenerative diseases^18^,^19^,^20^,^21^,^22^,^23^,^24^ largely through the secretion of inflammatory cytokines and other modulatory factors that affect neuronal survival and function. In particular, A1 reactive astrocytes, marked by elevated expression of complement component 3 (C3) and pro-inflammatory cytokines such as IL-6 and CXCL1, have been identified as contributors to synaptic dysfunction and neuronal loss^12^.

While astrocyte reactivity has been implicated in a wide range of neurodegenerative disorders, the molecular mechanisms that link mitochondrial dysfunction to astrocyte transformation and altered neuron-glia interactions remain unclear. In the context of POLG-related mitochondrial disease, it is not yet known whether intrinsic mitochondrial defects in astrocytes are sufficient to reprogram their identity and function, thereby contributing to disease progression. However, whether mitochondrial dysfunction directly drives astrocyte reactivity-and how this contributes to neuronal injury in *POLG* disease-remains largely unexplored. Recent work from our group has established that astrocytes derived from *POLG* patient iPSCs adopt a reactive A1-like phenotype, impaired mitochondrial respiration, and compromised neuronal support capacity^25^. These findings suggest that astrocyte-intrinsic mitochondrial defects may play a non-cell-autonomous role in promoting neurodegeneration. Nonetheless, the upstream triggers and downstream consequences of this transformation remain unclear.

Advancements in stem cell technology, such as induced pluripotent stem cells (iPSCs) and organoid models^24^,^26^,^27^,^28^, have enhanced our understanding of diseases such as POLG-related disorders. iPSC-derived models have shown mitochondrial dysfunction and mtDNA depletion typical of POLG disease^26^,^27^,^28^. Studies have also revealed that metabolic changes in astrocytes can induce a reactive, neurotoxic state. Our research found that iPSC-derived astrocytes from *POLG* mutation carriers exhibit metabolic deficits and secrete cytokines causing neuronal damage^29^.

In this study, we expand on these findings by employing an integrated model combining iPSC-derived cortical organoids, iPSC-derived astrocytes, single-cell RNA sequencing (scRNA-seq), and astrocyte-dopaminergic neuron co-cultures to comprehensively investigate the impact of *POLG* mutations on astrocyte identity and function. Our analyses reveal that *POLG* mutant astrocytes exhibit transcriptional profiles characteristic of A1 reactivity, elevated secretion of CXCL1, GROα, and IL-6, and loss of neurotrophic support. These changes are associated with significant transcriptomic remodeling in co-cultured neurons, including repression of genes involved in synaptic activity, cytoskeletal organization, and mitochondrial functions. Importantly, we identify nicotinamide riboside (NR), a NAD⁺ precursor, as a potential therapeutic strategy to mitigate astrocyte reactivity. NR treatment reduced inflammatory signaling, restored mitochondrial gene expression, and preserved neuronal integrity-findings consistent with previous studies showing NAD⁺ supplementation improves mitochondrial function and slows aging-related decline. Together, our work reveals a crucial role for mitochondrial integrity in controlling astrocyte fate and highlights the therapeutic potential of NAD⁺ metabolism modulation in *POLG*-related and other mitochondrial neurodegenerative diseases.

## Materials and methods

### Ethics approval

The project was approved by the Western Norway Committee for Ethics in Health Research (REK nr. 2012/919).

### Derivation of cortical organoids

Human iPSCs were reprogrammed from dermal fibroblasts of healthy donors and three patients carrying pathogenic *POLG* variants. The first line, WS5A, was homozygous p.W748S/p.W748S (c.2243G>C). The second line, CP2A, was compound heterozygous for the POLG variants p.A467T and p.W748S (c.1399G>A and c.2243G>C, respectively). The third line, the Alpers' line, was compound heterozygous for p.A467T and p.P589L (c.1399G>A and c.1766C>T, respectively). Isogenic control and healthy-donor iPSC lines were included throughout the study. Characterization of iPSCs, including assessment of pluripotency markers, karyotype integrity, and differentiation potential, were performed according to established protocols described in our previous publications^25^-^30^.

Cortical organoids were generated using an established protocol^28^,^31^. Human iPSCs were cultured in Essential 8 (E8) medium for at least three days prior to differentiation. On Day 1, iPSCs were dissociated into single cells using Accutase (Life Technologies, A1110501) for 10 minutes at 37°C, then pelleted by centrifugation at 300 ×g for 3 minutes. Approximately 9,000 cells per well were plated in a 96-well ultra-low attachment plate (Thermo Fisher Scientific, 174925) in neural induction medium containing 50 μM ROCK inhibitor to promote embryoid body (EB) formation under rotary conditions (85 rpm). Medium was partially replaced on Days 2, 4, 6, and 8 with fresh neural induction medium (NIM), with ROCK inhibitor was included only on Day 2. On Day 10, EBs were transferred to 6-well ultra-low attachment plates and cultured in neural differentiation medium (NDM) without vitamin A. From Day 18, NDM was supplemented with vitamin A, and organoids were placed on an orbital shaker for long-term maturation. Medium was replaced every 3-4 days and further supplemented with BDNF and ascorbic acid.

### iPSC-derived astrocyte and neuron co-culture

Astrocytes and dopaminergic neurons were derived from iPSCs as described previously^27^,^29^. For co-culture experiments, astrocytes (either control or *POLG* mutant) were seeded in Transwell inserts and co-cultured with dopaminergic neurons for 20 days.

### NR treatment

Cortical organoids and astrocyte cultures were treated with NR, kindly provided by Dr. Evandro Fei Fang (University of Oslo, Norway). Based on preliminary dose-response experiments assessing mitochondrial membrane potential and cell viability, 0.5 mM NR was selected as the optimal concentration for subsequent experiments, consistent with our previous study^25^.

Cortical organoids were treated for up to two months, whereas astrocyte cultures were treated for 7 days prior to downstream analyses. NR was replenished with each media change.

### NR dose-response analysis

To evaluate the dose-dependent effects of NR on mitochondrial function and cell viability, control and POLG patient-derived neural cells were treated with increasing concentrations of NR (0, 0.05, 0.25, 0.5, 1, and 2 mM). For mitochondrial membrane potential measurements, cells were stained with 100 nM tetramethylrhodamine ethyl ester (TMRE; Thermo Fisher Scientific, T669) to assess mitochondrial membrane potential (MMP) and 250 nM MitoTracker Green FM (MTG; Thermo Fisher Scientific, M7514) to measure mitochondrial mass. The TMRE signal was normalized to MTG intensity to obtain TMRE/MTG ratios, providing a measure of mitochondrial membrane potential per mitochondrial mass. Fluorescence signals were quantified using flow cytometry (Sony ID7000) and normalized to untreated controls.

Cell viability was assessed using the MTT assay (Thermo Fisher Scientific, V-13154) following NR treatment. After incubation with MTT reagent, the resulting formazan crystals were solubilized and absorbance was measured spectrophotometrically. Cell viability was expressed as fold change relative to untreated cells (0 mM NR).

### Immunofluorescence and confocal imaging

Organoids and co-cultures were fixed in 4% paraformaldehyde, permeabilized with 0.3% Triton X-100, and blocked with 5% normal goat serum. Samples were incubated overnight with primary antibodies against SOX2 (1:100; Abcam, ab97959), MAP2 (1:1000; Abcam, ab5392), GFAP (1:500; Abcam, ab4674), β-tubulin III (TUJ1) (mouse, 1:1000; Abcam, ab78078), Synaptophysin (1:500; Proteintech, 17785-1-AP), C3 (1:100; Abcam, ab181147), and S100A10 (1:100; Proteintech, CL488-66227), followed by appropriate Alexa Fluor-conjugated secondary antibodies. Imaging was performed using a Leica SP8 confocal microscope. Quantification of fluorescence intensity was performed using ImageJ (NIH).

### MitoTracker assays

Live iPSC-derived astrocytes were incubated with MitoTracker Green (MTG;Thermo Fisher Scientific, M7514, 500 nM) for 30 min at 37 °C in complete medium. Cells were washed three times with pre-warmed PBS and imaged live in phenol-red-free medium at 37 °C on a Leica STELLARIS confocal microscope (Leica Microsystems).

### Flow cytometry of A1 and A2 astrocyte markers

Cells were dissociated using Accutase (STEMCELL Technologies, 07920), washed with PBS, and stained with fluorescent-conjugated primary antibodies targeting C3 (Abcam, ab97462), GBP2 (Proteintech, 11854-1-AP), PTX3 (Proteintech, 13797-1-AP), GFAP (Abcam, ab7260), and S100A10 (Abcam, ab76472), all at 1:100 dilution. Staining was performed for 30 minutes on ice. Flow cytometric analysis was conducted using a Sony ID7000 spectral cell analyzer (Sony Biotechnology), and data were analyzed using FlowJo v10 (BD Biosciences). Median fluorescence intensity (MFI) was calculated and normalized. All experiments were performed in three independent biological replicates.

### Transmission electron microscopy (TEM)

Astrocytes were fixed in 2.5% glutaraldehyde in 0.1 M phosphate buffer, post-fixed in 1% osmium tetroxide, dehydrated through a graded ethanol series, and embedded in epoxy resin. Ultrathin sections (~70 nm) were stained with uranyl acetate and lead citrate, then imaged using a Hitachi HT7800 transmission electron microscope (Hitachi High-Tech, Japan) at 150,000× magnification. Mitochondrial morphology was assessed and quantified in randomly selected fields using ImageJ software (NIH).

### Western blot analysis

Total cellular proteins were extracted using RIPA lysis buffer supplemented with protease and phosphatase inhibitor cocktails. Protein concentrations were determined using a standard protein assay, and equal amounts of protein were separated by SDS-PAGE and transferred onto polyvinylidene difluoride (PVDF) membranes. Membranes were blocked with 5% non-fat milk or 5% BSA in TBST for 1 h at room temperature and then incubated overnight at 4 °C with primary antibodies against α-SMA (Abcam, ab7817), COXIV (Abcam, ab16056), TOMM20 (Abcam, ab56783), PGC-1α (Abcam, ab77210), NDUFB10 (Abcam, ab196019), Nestin (Abcam, ab22035), and GAPDH (Abcam, ab8245). To assess activation of the JAK-STAT signaling pathway, membranes were additionally probed with antibodies against phospho-STAT1 (Ser727) (Proteintech, 28977-1-AP), STAT1 (Proteintech, 101442-AP), phospho-STAT3 (Ser727) (Proteintech, 289451-AP), and STAT3 (Proteintech, 10253-AP). After incubation with HRP-conjugated secondary antibodies (Cell Signaling Technology, Cat. Nos. 7074 and 7076), protein bands were detected using an enhanced chemiluminescence (ECL) substrate (Thermo Fisher Scientific, SuperSignal™ West Pico PLUS, 34580) and visualized using a chemiluminescence imaging system. Band intensities were quantified using ImageJ software and normalized to GAPDH.

### Autophagic flux assay

Autophagic flux in iPSC-derived astrocytes was assessed using the DALGreen autophagy detection probe (Dojindo, D675), which selectively accumulates in acidic autophagic vesicles and enables monitoring of autophagic activity. Astrocytes were seeded in 12-well plates and cultured until approximately 70% confluency. Cells were then incubated with DALGreen (0.5 µM) in pre-warmed culture medium for 40 min at 37 °C to allow probe uptake. After staining, cells were washed with PBS. Autophagy was pharmacologically modulated using rapamycin (100 nM for 24 h) to induce autophagy and bafilomycin A1 (20 nM during the final 4 h) to inhibit lysosomal degradation. Four treatment conditions were included: vehicle control, rapamycin alone, bafilomycin A1 alone, and rapamycin combined with bafilomycin A1. Following treatment, cells were washed, dissociated using TrypLE Express (Gibco, 12604013), and analyzed by flow cytometry using 488 nm excitation with FITC-like emission detection. Median fluorescence intensity (MFI) was quantified and normalized to vehicle controls. An increase in DALGreen signal in the presence of bafilomycin A1 relative to rapamycin alone was interpreted as evidence of active autophagic flux. Experiments were performed using iPSC-derived astrocytes from control and POLG patient lines, with three independent biological replicates for each condition.

### mtDNA copy number quantification

Total DNA was extracted using a DNeasy kit (Qiagen, 69506). mtDNA and nuclear DNA were quantified by qPCR using primers targeting ND1 (mitochondrial) and APP (nuclear) genes. Copy number was calculated as the ratio of mtDNA to nuclear DNA.

### ScRNA-seq for astrocytes

Single-cell RNA sequencing (scRNA-seq) was performed using astrocytes derived from two independent healthy control iPSC lines, two WS5A patient-derived iPSC clones, and two CP2A patient-derived iPSC clones. Single-cell suspensions were generated by enzymatic dissociation of iPSC-derived astrocytes. Cell viability and concentration were assessed using 0.4% (w/v) Trypan Blue solution (Gibco, 15250-061). Cell numbers and viability were determined using a hemocytometer under a light microscope. Only samples with high viability were used for downstream scRNA-seq analysis.

### ScRNA-seq library preparation and data processing

Single-cell suspensions were prepared from iPSC-derived astrocytes and processed for scRNA-seq using the GEXSCOPE Single Cell RNA-seq Library Kit (Singleron Biotechnologies, 4161031) according to the manufacturer's instructions. Briefly, cells were resuspended in PBS containing 0.04% BSA and adjusted to a final concentration of 3 × 10⁵ cells/mL. Approximately 6,000 cells per sample were captured using a microfluidic chip. Paramagnetic beads conjugated with oligo(dT) probes containing unique molecular identifiers (UMIs) and cell barcodes were used to capture polyadenylated mRNA molecules. The captured mRNA was reverse transcribed into cDNA and amplified by PCR. Amplified cDNA was subsequently fragmented and ligated with indexed Illumina sequencing adapters to generate sequencing libraries.

Library fragment size distribution was assessed using an Agilent Fragment Analyzer, and libraries were quantified using a Qubit 4.0 fluorometer (Thermo Fisher Scientific). Sequencing was performed on an Illumina NovaSeq 6000 platform (Illumina, San Diego, CA, USA) using a paired-end 2 × 150 bp configuration, generating approximately 90 Gb of sequencing data per library. Sequencing reads were demultiplexed based on sample indices using the Illumina BaseSpace Cloud platform.

Raw sequencing data were processed using CeleScope software (v1.3.0; Singleron Biotechnologies; https://github.com/singleron-RD/CeleScope) with default parameters. Low-quality reads were removed and the remaining reads were aligned to the human reference genome (GRCh38) using STAR. Gene annotation was performed using Ensembl release 92, and gene expression quantification was conducted using featureCounts, generating a gene-cell count matrix containing the number of unique molecular identifiers (UMIs) per gene in each cell.

Downstream analyses were performed using the Scanpy package in Python. Quality control metrics, including the number of detected genes per cell (nFeature_RNA) and the percentage of mitochondrial transcripts (percent_mt), were calculated using the calculate_qc_metrics function. Cells with > 20% mitochondrial gene expression or > 5,000 detected genes (suggesting potential doublets) were excluded, while cells with < 200 detected genes, indicative of cell debris, were also removed prior to downstream analyses.

To minimize technical variation across samples, all astrocyte lines were processed using the same library preparation protocol and sequencing platform. Data were normalized and scaled prior to dimensionality reduction and clustering, and cells from different lines were integrated for downstream comparative analyses.

### Bulk RNA sequencing

For transcriptomic analysis of dopaminergic neurons co-cultured with astrocytes, total RNA was extracted using TRIzol reagent according to the manufacturer's instructions. RNA quality and integrity were assessed using an Agilent 2100 Bioanalyzer. Astrocytes used in the co-culture experiments were derived from two healthy control iPSC lines and two POLG patient-derived lines (WS5A and CP2A). For each genotype, astrocytes from two independent iPSC clones were pooled prior to co-culture to minimize clone-specific variability. Control astrocytes were similarly pooled from two independent control lines. Library preparation and high-throughput sequencing were performed by BGI Genomics (Shenzhen, China) using the Illumina NovaSeq 6000 platform. Paired-end reads (150 bp) were generated and subjected to quality control, filtering, and adapter trimming.

Bioinformatic processing, including alignment to the human reference genome (GRCh38) using STAR, and quantification of gene expression levels, was performed by BGI. Differential gene expression analysis was conducted using DESeq2, and genes with an adjusted p-value (FDR) < 0.05 were considered significantly differentially expressed. Downstream enrichment analysis of DEGs was performed using GSEA and the KEGG pathway database.

### Statistical analysis

All statistical analyses were performed using GraphPad Prism 9 (GraphPad Software, San Diego, CA, USA). Data are presented as mean ± standard error of the mean (SEM). Comparisons between POLG and control groups were performed using the nonparametric Mann-Whitney U test. For comparisons between NR-treated and untreated samples from the same cell lines, paired two-tailed Student's t-tests were used. For comparisons involving more than two groups, one-way analysis of variance (ANOVA) followed by appropriate post hoc tests was performed. For analysis involving repeated measurements across markers in astrocytes derived from different patient lines, a two-way mixed-effects model (REML) followed by Tukey's multiple comparisons test was used. A p-value < 0.05 was considered statistically significant unless otherwise specified.

## Results

### Generation and characterization of iPSC-derived brain organoids

In our previous work^25^, we established an iPSC-derived astrocyte model to study the pathological impact of *POLG* mutations on astrocyte function. We demonstrated that *POLG* mutant astrocytes adopted a reactive, neurotoxic phenotype, characterized by elevated expression of the A1 astrocyte marker C3 and reduced levels of the A2 neuroprotective marker S100A10. Functional assays further showed that these astrocytes exerted deleterious effects on neuronal survival in co-culture, suggesting a critical role for astrocyte-mediated toxicity in the pathogenesis of POLG-related neurodegeneration.

**Figure 1:**
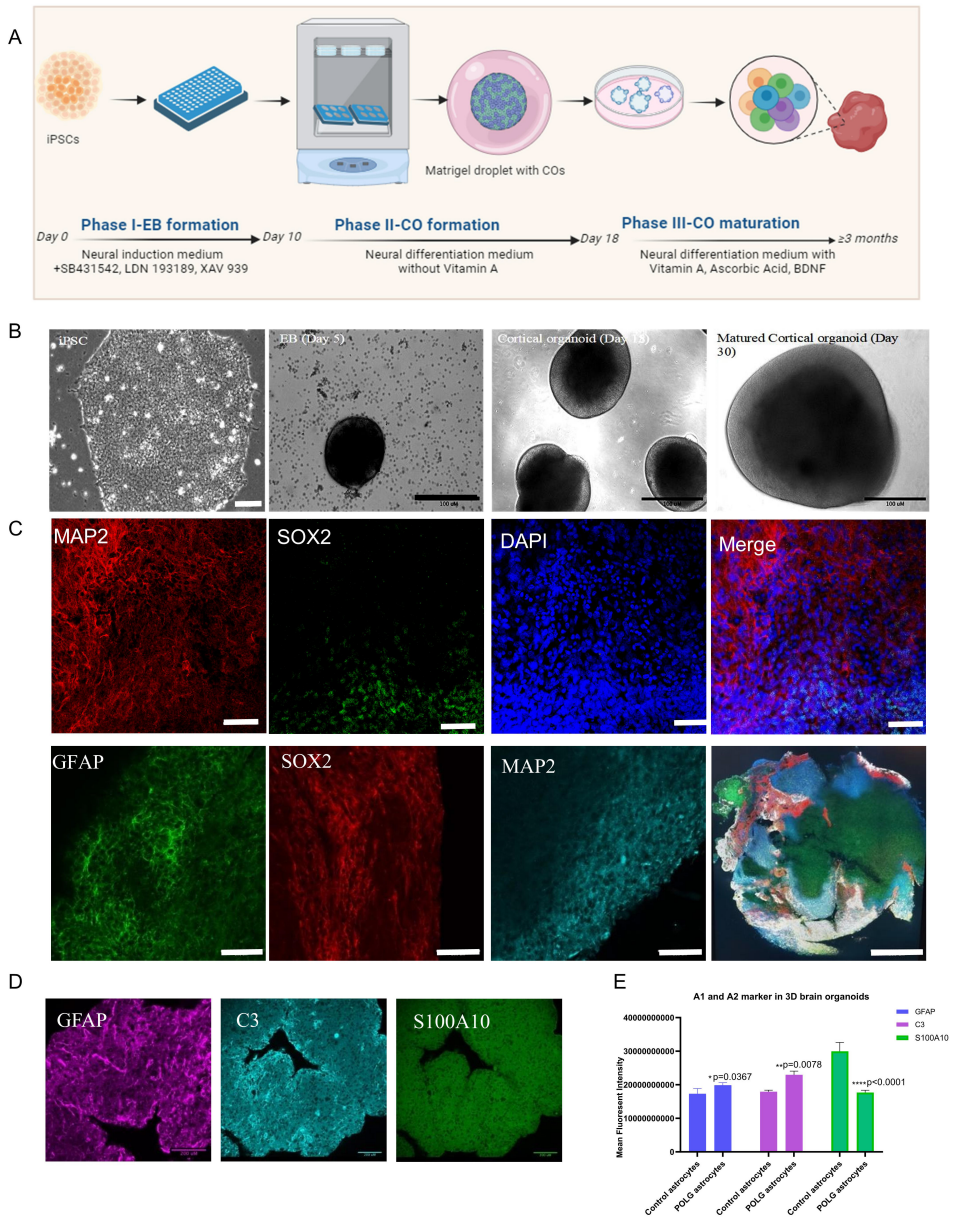
** Generation and characterization of POLG and healthy control iPSC-derived cortical organoids. (A)** Schematic illustration of the three-phase cortical organoid differentiation protocol. Phase I (Day 0-10): EB formation using dual SMAD inhibition (SB431542, LDN193189) and WNT inhibition (XAV939). Phase II (Day 10-18): neural induction and Matrigel embedding. Phase III (Day ≥18): long-term maturation in differentiation medium supplemented with Vitamin A, ascorbic acid, and BDNF. **(B)** Brightfield images showing representative stages of differentiation from iPSCs (Day 0), EBs (Day 5), early cortical organoids (Day 18), and matured cortical organoids (Day 30). The scale bar is 100 μm (black) and 50 μm (white). **(C)** Immunofluorescence staining of Day 30 cortical organoids showing the presence of MAP2+ neurons (red), SOX2+ progenitors (green), and DAPI+ nuclei (blue). Merged image confirms the layered organization of neural populations. The scale bar is 200 μm. **(D)** High-resolution immunostaining showing increased GFAP (magenta) and A1 reactive astrocyte marker C3 (cyan), and decreased A2 marker S100A10 (green) in POLG organoids. **(E)** Quantification of fluorescence intensity for GFAP, C3, and S100A10 (normalized to DAPI), demonstrating enhanced A1 reactivity and reduced A2 phenotype in POLG WS5A astrocytes. Data are presented as mean ± SEM from independent biological replicates. Statistical significance was determined using the Mann-Whitney U test (*p* < 0.05; **p** < 0.01; ns, not significant). POLG astrocytes were derived from the WS5A patient line. At least three independent experiments were performed.

Building on these findings, we developed a 3D cortical organoid system derived from human iPSCs to more comprehensively examine the cellular and molecular consequences of POLG astrocyte dysfunction within a human neural context. As shown in Figure 1A (top panel), a stepwise differentiation protocol was employed. Phase I involved EB formation using dual SMAD and WNT pathway inhibition (SB431542 and XAV939). Phase II facilitated neuroectodermal patterning and Matrigel embedding, while Phase III supported long-term maturation with supplements including Vitamin A, ascorbic acid, and BDNF. Brightfield imaging confirmed progressive morphogenesis from iPSC colonies (Day 0) to EBs (Day 5), early neural organoids (Day 18), and mature cortical structures by Day 30 (Figure 1B).

Immunofluorescence staining validated successful neural lineage specification (Figure 1C). Mature organoids contained SOX2+ neural progenitor zones, MAP2+ post-mitotic neurons, and GFAP+ astrocytes. 3D reconstruction further demonstrated appropriate laminar organization and spatial segregation of neural subtypes within the cortical architecture. Consistent with our previous study in POLG cerebral organoids^28^, quantitative analysis of neuronal markers demonstrated a reduction in neuronal populations in *POLG* mutant organoids.

To investigate astrocyte reactivity within the organoid system, we examined the expression of key astrocytic markers, including GFAP (a general astrocyte reactivity marker), C3 (indicative of the neuroinflammatory A1 state), and S100A10 (a hallmark of the neuroprotective A2 phenotype) (Figure 1D). Quantitative immunostaining and fluorescence intensity analysis revealed a significant upregulation of GFAP and C3, alongside a marked reduction in S100A10, in *POLG* mutant cortical organoids compared to controls (Figure 1E). These results reinforce our previous observations and suggest that *POLG* mutations induce a sustained reactive astrocyte state in the organoid context.

Together, these results demonstrate that *POLG* mutations not only induce a reactive astrocyte phenotype in 3D models but also drive a loss of neuroprotective A2 astrocytes and neuronal populations.

### POLG iPSC-derived astrocytes exhibit mitochondrial dysfunction, reactive A1 polarization, and altered STAT signaling

**Figure 2:**
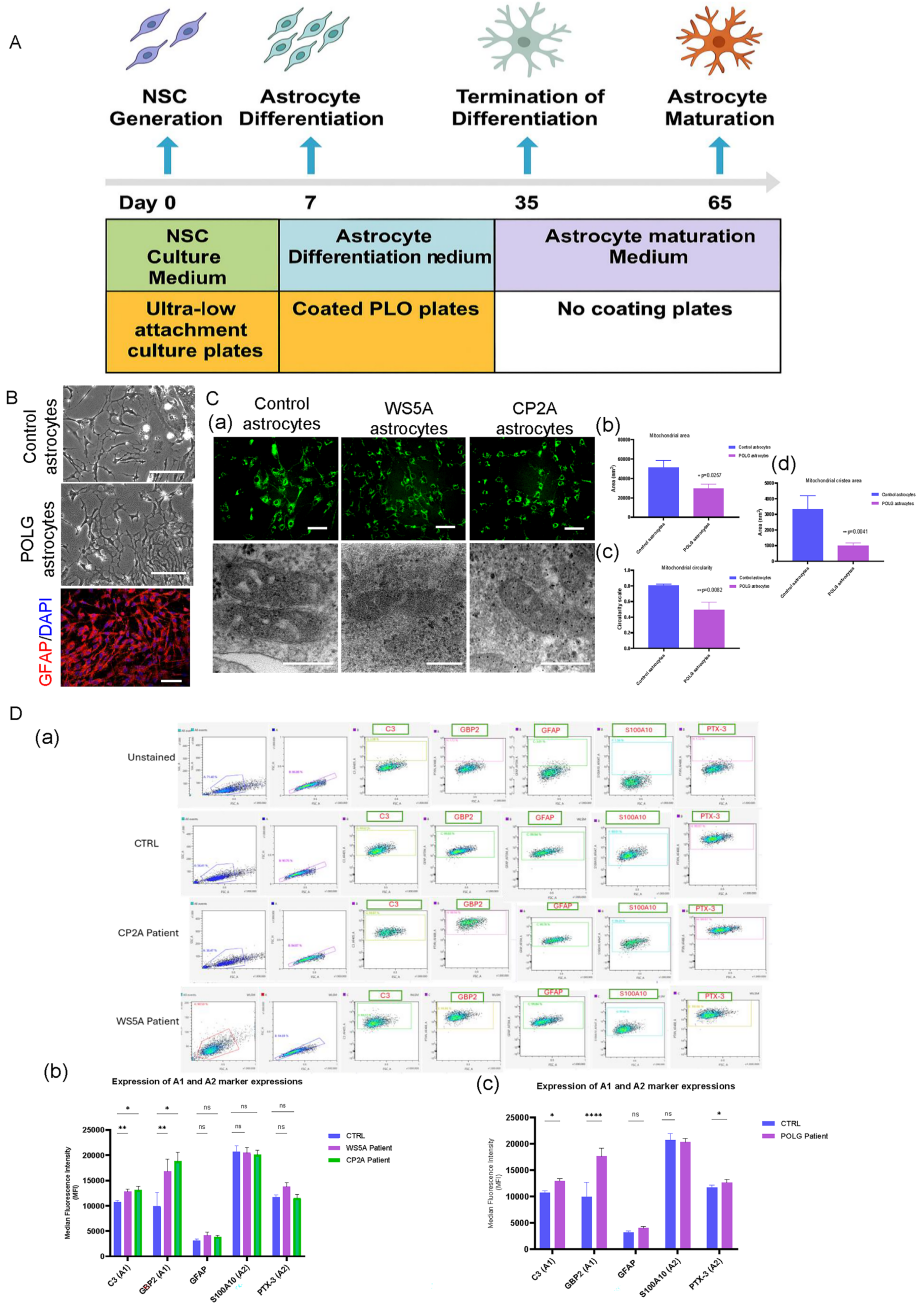
** Characterization of POLG iPSC-derived astrocytes reveals a reactive phenotype and enhanced mitophagy. (A)** Schematic of the astrocyte differentiation protocol from human iPSCs. Neural stem cells (NSCs) were generated by Day 5 in NSC culture medium and cultured in ultra-low attachment plates. From Day 7, astrocyte differentiation was initiated in coated PLO plates using astrocyte differentiation medium. Differentiation was terminated on Day 35, and cells were subsequently matured in astrocyte maturation medium on uncoated plates until Day 65. **(B)** Representative MitoTracker Green (MTG) fluorescence and transmission electron microscopy (TEM) images illustrating mitochondrial morphology in control, WS5A, and CP2A patient-derived astrocytes (a). Quantitative analysis of mitochondrial area (b), circularity (c), and cristae area (d). Scale bars: MTG, 100 µm; TEM, 100 nm. Data are presented as mean ± SEM from independent biological replicates. Statistical significance was determined using the Mann-Whitney U test. *p < 0.05; **p < 0.01; ns, not significant. POLG astrocytes were derived from both WS5A and CP2A patient lines. At least three independent experiments were performed. **(C)** Flow cytometry analysis and quantification of reactive astrocyte markers. (a) Representative dot plots showing increased expression of A1 markers (C3, GBP2), A2 marker (PTX3, S100A10), and GFAP in POLG astrocytes compared to controls. (b) Quantification of marker expressions in astrocytes derived from WS5A and CP2A POLG patient lines (WS5A and CP2A). Data represent mean ± SEM from at least three independent experiments; statistical significance was performed using a two-way mixed-effects model (REML) followed by Tukey's multiple comparisons test. *p < 0.05; **p < 0.01; ns, not significant. At least three independent experiments were performed.

To investigate the effects of *POLG* mutations on astrocyte reactivity and mitochondrial dynamics, we established a long-term differentiation protocol to generate astrocytes from *POLG*-mutant and isogenic control iPSCs (Figure 2A). Phase-contrast imaging revealed that control astrocytes displayed typical stellate morphology with extended processes, while POLG-derived astrocytes appeared more elongated and flattened, features suggestive of a reactive-like state (Figure 2B, upper and middle panels). Immunofluorescence staining confirmed robust expression of glial fibrillary acidic protein (GFAP, red) with nuclear DAPI (blue) in both control and POLG astrocytes, validating their astrocytic identity (Figure 2B, lower panel). MTG staining revealed a clear alteration in mitochondrial distribution and morphology in both WS5A and CP2A POLG astrocytes, characterized by fragmented and punctate mitochondrial structures compared to the elongated and interconnected mitochondrial networks seen in controls (Figure 2C, a, upper panel). These observations indicate impaired mitochondrial dynamics and potential mitochondrial stress in POLG astrocytes. TEM further confirmed significant ultrastructural mitochondrial abnormalities in both WS5A and CP2A POLG astrocytes, including fragmented mitochondria, disrupted or sparse cristae, and electron-lucent areas, consistent with mitochondrial damage and functional impairment (Figure 2C, a, lower panel). Quantitative analysis confirmed significantly reduced mitochondrial area (Figure 2C, b), circularity (Figure 2C, c), and cristae area (Figure 2C, d) in POLG astrocytes compared with controls.

Next, we quantified A1/A2 markers by flow cytometry in POLG and isogenic control astrocytes to assess genotype-dependent effects (Figure 2D). We analyzed two patient-derived iPSC lines with distinct mutations: WS5A (p.W748S/W748S) and CP2A (p.A467T/W748S). At baseline, POLG astrocytes showed significantly higher A1 markers (C3, GBP2) and lower A2 markers (S100A10, PTX3) compared with controls, and the shift was more pronounced in WS5A than CP2A (Figure 2D, b), consistent with a pro-inflammatory, neurotoxic state. GFAP levels were unchanged, indicating preserved astrocyte identity despite altered reactivity. Upon NR treatment, pooled analysis across both POLG lines demonstrated a significant suppression of A1 markers and a selective increase in the A2 marker PTX3, with no detectable change in S100A10 (Figure 2D, c).

To investigate whether inflammatory signaling pathways are altered in POLG astrocytes, we examined the activation status of the STAT3 and STAT1 pathways by Western blot analysis. Quantification of total protein levels revealed that STAT3 expression was significantly increased in POLG astrocytes compared with control astrocytes, whereas total STAT1 expression showed no significant difference between groups (Figure S1). Although the absolute levels of phosphorylated STAT3 (p-STAT3, Ser727) and phosphorylated STAT1 (p-STAT1, Ser727) did not show statistically significant differences when normalized to GAPDH, an analysis of phosphorylation ratios revealed differential regulation of STAT signaling. Specifically, the p-STAT3/STAT3 ratio did not show a significant change between control and POLG astrocytes, whereas the p-STAT1/STAT1 ratio was significantly reduced in POLG astrocytes, indicating altered STAT signaling dynamics.

Collectively, these findings demonstrate that *POLG* mutations promote astrocyte reactivity characterized by mitochondrial dysfunction, shift toward a neurotoxic A1 phenotype, and alter STAT1 signaling.

### Single-cell transcriptomics uncovered distinct reactivity and comprehensive profiling of A1 astrocytes in POLG astrocytes under neuroinflammatory conditions

**Figure 3:**
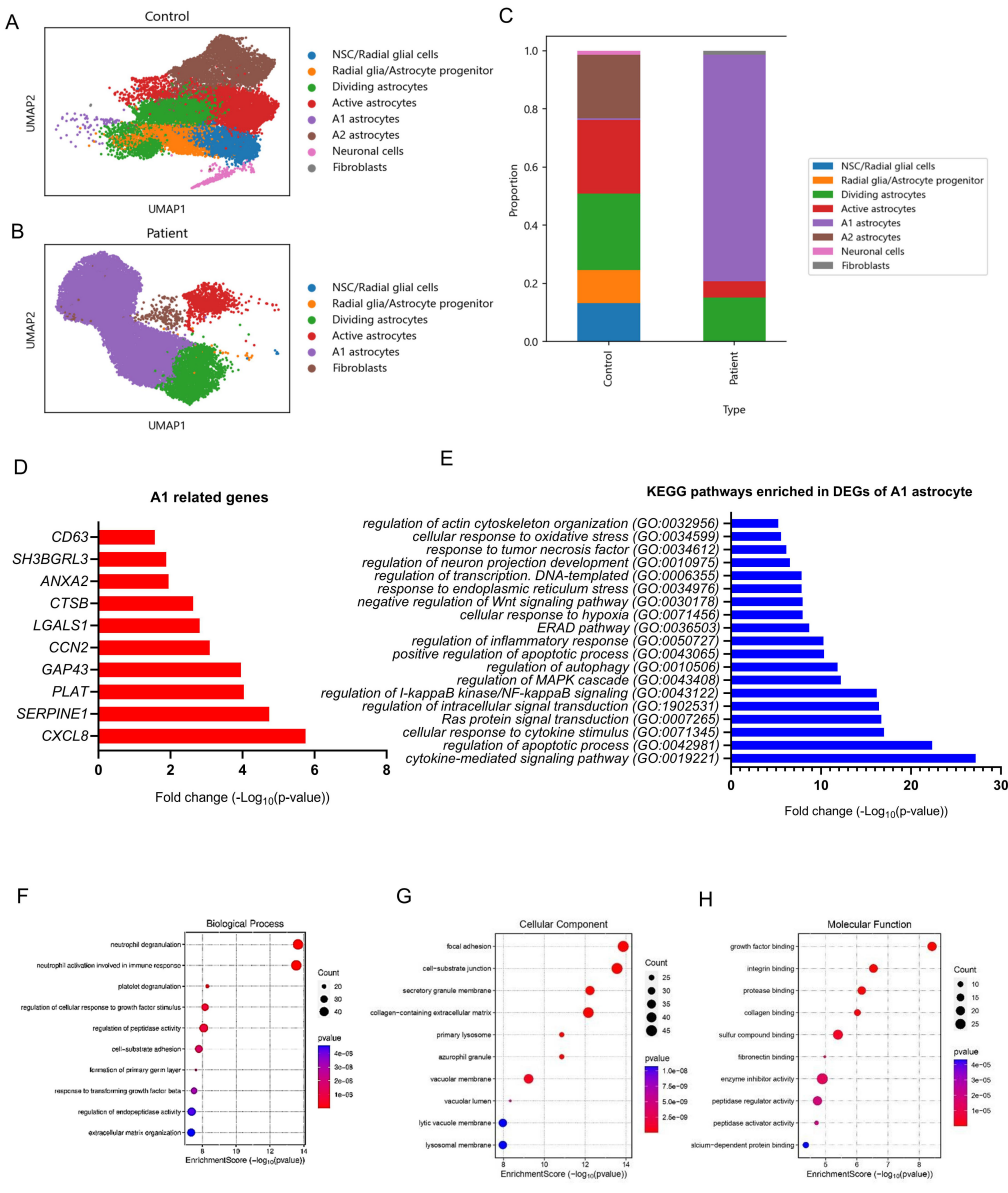
** Single-cell transcriptomic analysis reveals expansion of reactive A1 astrocytes and inflammatory pathway activation in POLG astrocytes. (A, B)** UMAP plots showing cell clustering of healthy control (A) and *POLG* mutant (B) astrocytes. Cell identities were annotated based on canonical marker expression. **(C)** Proportional bar plot comparing cell-type composition between healthy control and WS5A and CP2A astrocytes. POLG astrocytes show a marked expansion of A1 astrocytes and reduction of A2 astrocytes and neurons. **(D)** Bar plot showing upregulation of representative A1-specific genes in POLG astrocytes, including CXCL8, SERPINE1, GAP43, and others. **(E)** KEGG pathway enrichment analysis of DEGs in A1 astrocytes from POLG patients, highlighting activation of TGF-β signaling, autophagy, actin cytoskeleton regulation, along with downregulation of oxidative phosphorylation pathways. **(F-H)** GO enrichment analysis of DEGs in A1 astrocytes categorized by: **(F)** GO: BP (e.g., neutrophil degranulation, ECM organization), **(G)** GO: CC (e.g., lysosomal membrane, focal adhesion), and **(H)** GO: MF (e.g., protease and integrin binding). Dot size reflects gene count; color indicates p-value. ScRNA-seq was performed using astrocytes derived from two independent healthy control iPSC lines, two WS5A patient-derived iPSC clones, and two CP2A patient-derived iPSC clones.

To further examine the impact of *POLG* mutations on cell fate determination within the iPSC-derived astrocyte model, we performed scRNA-seq followed by UMAP clustering of cells derived from isogenic control and *POLG* mutant astrocytes. In isogenic control astrocytes (Figure 3A), we identified diverse neural and glial cell populations, including neural stem cells/radial glial cells, radial glia/astrocyte progenitors, dividing astrocytes, active astrocytes, A1 astrocytes, A2 astrocytes, neuronal cells, and a small population of fibroblast-like cells. The distribution of astrocyte subtypes appeared relatively balanced, with both A1 and A2 populations present in similar proportions. In contrast, *POLG* mutant astrocytes (Figure 3B) exhibited a striking remodeling of the cellular composition. There was a marked expansion of A1 astrocytes and a loss of A2 astrocytes, alongside an overall enrichment of astrocyte-lineage cells.

The stacked bar plot (Figure 3C) revealed clear differences in lineage allocation. In healthy control astrocytes, the cell population was relatively heterogeneous, with contributions from NSC/radial glial cells, radial glia-astrocyte progenitors, neuronal cells, as well as a balanced distribution of A1, A2, and active astrocytes. This suggests a healthy neuro-glial developmental trajectory. In contrast, POLG astrocytes were dominated by A1 astrocytes, which comprised the majority of astroglial populations. The proportion of A2 astrocytes was dramatically reduced, consistent with previous immunofluorescence and UMAP findings. Additionally, the neuronal cell population was substantially decreased, while fibroblast-like cells showed a slight increase. Notably, early progenitor populations such as NSCs and dividing astrocytes were largely depleted in POLG astrocytes, indicating potential premature astrocytic commitment and impaired neurogenesis.

To further validate the enrichment of reactive A1 astrocytes at the molecular level, we analyzed the expression of canonical A1-associated genes in the scRNA-seq dataset. As shown in Figure 3D, multiple A1 markers were significantly upregulated in *POLG* mutant astrocytes compared to healthy controls. Among the most highly induced genes were *CXCL8*, *SERPINE1*, *PLAT*, and *GAP43*, all of which are known mediators of inflammation, reactive gliosis, or neurotoxicity. Other A1 markers such as *CCN2*, *LGALS1*, and *CTSB* also exhibited substantial upregulation, further supporting the presence of a robust A1 signature. The modest increase in *CD63*, *ANXA2*, and *SH3BGRL3* suggests that both core and accessory A1 gene modules are activated in the POLG context.

To explore the molecular pathways underlying A1 astrocyte reactivity in POLG astrocytes, we performed KEGG pathway enrichment analysis on DEGs derived from A1 astrocyte clusters. Based on enrichment analysis of DEGs from A1 astrocyte clusters, we identified significant upregulation of pathways involved in stress, inflammation, and apoptotic regulation (Figure 3E and Table S2). Notably enriched biological processes included cytokine-mediated signaling, cellular response to cytokines, and regulation of apoptotic processes, highlighting a strong pro-inflammatory and stress-responsive phenotype. Pathways such as NF-κB signaling, MAPK cascade, and autophagy regulation further suggest active intracellular signaling reprogramming. Additional enrichment in ER stress response, ER-associated degradation (ERAD), and response to oxidative and hypoxic stress indicates disrupted proteostasis and mitochondrial stress. Moreover, regulation of transcription, neuron projection development, and actin cytoskeleton organization suggest possible impacts on neuro-glial communication and structural remodeling.

To gain further insight into the biological functions altered in A1 astrocytes from *POLG* mutant astrocytes, we conducted Gene Ontology (GO) enrichment analysis focused on the Biological Process (BP) category. As shown in Figure 3F, the most significantly enriched processes included neutrophil degranulation, neutrophil activation involved in immune response, and platelet degranulation, terms typically associated with inflammatory and innate immune signaling. These results suggest that A1 astrocytes in *POLG*-mutant astrocytes may adopt immune-like characteristics that enhance their pro-inflammatory behavior. Additional enriched pathways such as regulation of cellular response to growth factor stimulus, cell-substrate adhesion, and extracellular matrix organization further indicate that A1 astrocytes are actively remodeling their environment, potentially affecting both astrocyte-neuron interactions and tissue structure. The enrichment of TGF-β response pathways, consistent with the KEGG analysis, supports a role for this signaling axis in promoting A1 astrocyte reactivity.

To better understand the subcellular localization of upregulated gene products in A1 astrocytes, we performed GO enrichment analysis for Cellular Component (CC) terms. The analysis revealed significant enrichment of components involved in vesicular activity, adhesion, and matrix interaction (Figure 3G). Highly enriched terms included focal adhesion and cell-substrate junction, suggesting enhanced adhesion capacity or remodeling of cell-matrix interactions-hallmarks of reactive astrocyte behavior. In addition, enrichment in secretory granule membranes, azurophilic granules, and lysosomal components (including vacuolar membrane, lysosomal membrane, and primary lysosome) indicates an upregulation of vesicle-mediated secretion and degradation pathways, consistent with inflammatory cytokine release and increased phagolysosomal activity.

To complement pathway and localization analyses, we conducted GO enrichment for Molecular Function (MF) terms based on A1 astrocyte-specific DEGs. As shown in Figure 3H, the most significantly enriched functions included growth factor binding, integrin binding, protease binding, and collagen binding, all of which are key components of cell-matrix interactions, trophic factor signaling, and inflammatory responses. These functions suggest that A1 astrocytes may engage in aberrant signaling with the extracellular matrix and surrounding neurons, potentially promoting tissue remodeling or degeneration. Additional enriched terms, such as enzyme inhibitor activity and peptidase regulator activity, point to a reprogramming of proteolytic control within these cells, consistent with inflammatory or reactive phenotypes. The enrichment of calcium-dependent protein binding may further reflect changes in intracellular signaling and excitability.

Next, we mapped differentially expressed genes (DEGs) from A1 astrocytes to the oxidative phosphorylation (OXPHOS) pathway using KEGG pathway visualization (Figure S2). The analysis revealed significant transcriptional downregulation in multiple components of mitochondrial respiratory chain complexes, particularly within Complex I (NADH dehydrogenase) and Complex IV (cytochrome c oxidase). Notably, several subunits of Complex I such as *NDUFS4, NDUFS6, NDUFA10*, and *NDUFB10* were downregulated, suggesting impaired electron transfer and redox activity. Additionally, core components of Complex III (e.g., *UQCRC1*) and ATP synthase subunits in Complex V were also affected, with decreased expression of *ATP5F1E* and *ATP5MC3*, indicating potential compromise in ATP production capacity. These transcriptomic alterations highlight a global suppression of oxidative phosphorylation machinery in reactive A1 astrocytes, supporting the hypothesis that mitochondrial dysfunction contributes to their neurotoxic phenotype.

Given the significant enrichment of the TGF-β signaling pathway in KEGG pathway analysis, we mapped differentially expressed genes from A1 astrocytes to the TGF-β pathway using KEGG visualization (Figure S3). The analysis revealed upregulation of key components of the canonical TGF-β signaling cascade, including *TGFB1*, *TGFBR1*, and the downstream effectors *SMAD2, SMAD3*, and *SMAD7*. Additionally, transcriptional targets such as *FOS, ID1*, and *ZEB2* were also elevated, suggesting active TGF-β-mediated transcriptional regulation.

To further validate the enrichment of autophagy-related pathways observed in KEGG analysis (Figure S4), we mapped differentially expressed genes from A1 astrocytes onto the KEGG Autophagy - animal pathway. As illustrated in Figure S4, multiple key regulators of autophagy was upregulated in A1 astrocytes, including *BECN1, ATG3, ATG7, MAP1LC3B*, and *GABARAPL1*. These genes represent critical nodes in the initiation, elongation, and maturation phases of autophagosome formation, suggesting that autophagic flux is enhanced in A1-reactive astrocytes. This may reflect an adaptive or pathological response to cellular stress, mitochondrial dysfunction, or accumulated protein aggregates within the astrocytic cytoplasm.

To determine whether these transcriptional changes were associated with functional alterations in autophagy, we measured autophagic flux using a DALGreen-based fluorescence assay (Figure S5). Autophagy-lysosome activity was assessed using the DALGreen probe, which selectively accumulates in acidic autolysosomes. Under basal conditions, POLG astrocytes (WS5A and CP2A) exhibited significantly reduced DALGreen fluorescence compared with control astrocytes, indicating decreased basal autophagy-lysosome activity. Treatment with rapamycin significantly increased DALGreen fluorescence in all groups, suggesting that the autophagy pathway remains responsive to pharmacological induction. In contrast, bafilomycin A1 treatment markedly reduced DALGreen fluorescence due to inhibition of lysosomal acidification, which is required for DALGreen signal generation. Under conditions of lysosomal inhibition (bafilomycin or rapamycin + bafilomycin), differences between control and POLG astrocytes were no longer observed. Together, these results indicate that POLG astrocytes display reduced basal autophagy-lysosome activity while retaining the capacity for pharmacological induction of autophagy.

These findings suggest that *POLG* mutations lead to a marked expansion of neurotoxic A1 astrocytes and a reduction in neurons and A2 astrocytes. Transcriptomic analyses showed activation of inflammatory, TGF-β, autophagy, and metabolic stress pathways in POLG A1 astrocytes. These findings indicate that *POLG* mutations drive astrocyte reprogramming toward a reactive, pro-inflammatory state that may contribute to neuronal loss.

### POLG patient A1 astrocytes showed disruptions of neural and mitochondrial functions

**Figure 4:**
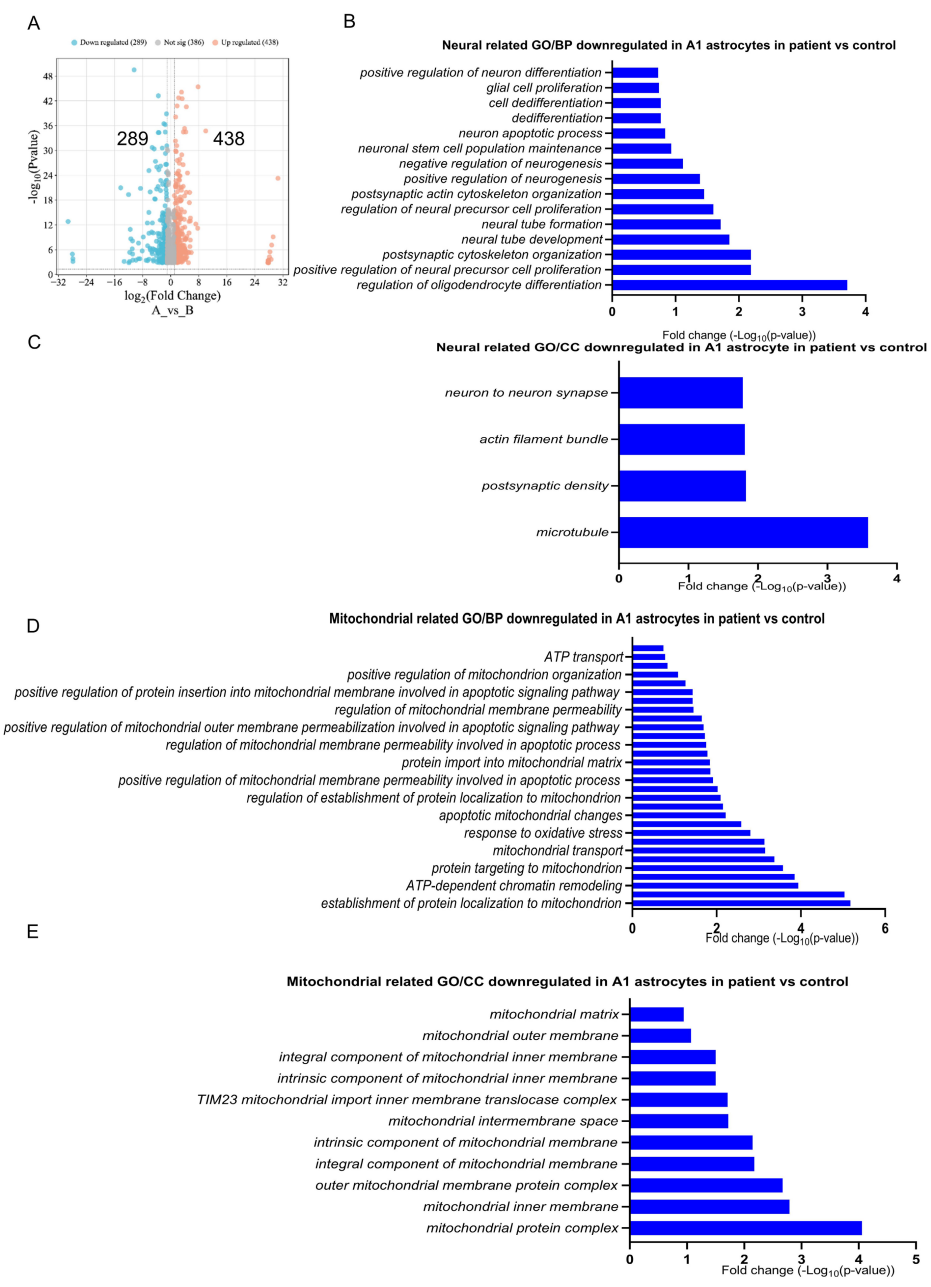
** Transcriptomic analysis reveals widespread downregulation of neural, mitochondrial, and immune-related genes in patient-derived A1 astrocytes. (A)** Volcano plot showing DEGs in A1 astrocytes from POLG patient-derived astrocytes versus healthy controls. A total of 438 genes were upregulated (red) and 289 downregulated (blue). **(B)** GO BP terms related to neural development were significantly downregulated in POLG A1 astrocytes, including pathways related to neurogenesis, neural stem cell maintenance, and postsynaptic organization. **(C)** Neural-related GO CC terms such as postsynaptic density, microtubule, and synaptic structure were also reduced, pointing to impaired astrocyte-neuron interaction and cytoskeletal remodeling. **(D)** GO BP enrichment of downregulated mitochondrial processes highlights impaired ATP transport, mitochondrial membrane organization, import machinery, and oxidative stress response. **(E)** GO CC terms related to mitochondrial structure were significantly downregulated, including mitochondrial matrix, inner membrane, and TIM23 import system, indicating disruption of mitochondrial integrity.

Our analysis using a volcano plot showed distinct patterns of gene expression between the two groups. We identified 438 upregulated genes and 289 downregulated genes in patient A1 astrocytes versus control, while 386 genes exhibited no significant change (Figure 4A). To investigate the functional impairments of A1 astrocytes in the context of *POLG* mutation, we performed GO BP enrichment analysis on downregulated genes specifically in the A1 astrocyte cluster from POLG patient-derived astrocytes, relative to healthy controls. As shown in Figure 4B and Table S3, many of the most significantly downregulated GO terms were associated with neuronal development, neurogenesis, and neural precursor cell function. These included processes such as "positive regulation of neurogenesis", "regulation of neural precursor cell proliferation", "neural tube formation", and "postsynaptic cytoskeleton organization". In addition, astrocyte functions linked to glial proliferation, dedifferentiation, and regulation of oligodendrocyte differentiation were also suppressed.

To further explore the structural basis of neuro-supportive impairment in POLG A1 astrocytes, we examined downregulated GO CC terms associated with synaptic and cytoskeletal architecture. As shown in Figure 4C and Table S4, several key components were significantly reduced in POLG astrocytes. Specifically, terms such as “neuron to neuron synapse”, “postsynaptic density”, “actin filament bundle”, and “microtubule” were downregulated, suggesting that POLG A1 astrocytes may have limited interaction with neuronal structures, and impaired ability to support or modulate synapse formation and maintenance. The suppression of cytoskeletal and synaptic architecture components also aligns with observed reductions in astrocyte-neuron signaling and may reflect a diminished capacity for tripartite synapse formation, trophic support, or response to neuronal activity.

To investigate mitochondrial dysfunction in *POLG* mutant astrocytes, we performed GO enrichment analysis focused on downregulated mitochondrial-related biological processes (BP) in A1 astrocytes. As shown in Figure 4D and Table S5 & S6, a broad suppression of genes involved in mitochondrial structure, transport, and activity was observed. Key downregulated processes included ATP transport, mitochondrial membrane organization, protein import into the mitochondrial matrix, and oxidative stress response. Notably, terms such as positive regulation of mitochondrial function, mitochondrial transport, and protein targeting to mitochondria were also significantly reduced. These findings indicate a general repression of mitochondrial biogenesis, energy metabolism, and quality control pathways in POLG A1 astrocytes.

The mitochondrial-related GO CC analysis revealed significant downregulation of key mitochondrial structures in patient A1 astrocytes compared to controls (Figure 4E and Table S7). Notably, components essential for mitochondrial integrity and function, including the mitochondrial matrix, outer membrane, and intermembrane space, exhibited reduced expression. Furthermore, critical structural components such as the mitochondrial inner membrane, intrinsic and integral components of the mitochondrial membrane, and the *TIM23* mitochondrial inner membrane import system were also significantly downregulated, indicating potential impairments in mitochondrial transport and protein import. The suppression of outer mitochondrial membrane protein complexes and other mitochondrial protein complexes suggests disruptions in essential mitochondrial processes, which may affect energy production, metabolic regulation, and cellular resilience to stress. These findings further highlight mitochondrial dysfunction as a key feature of A1 astrocytes in patient samples, reinforcing its potential role in neurodegenerative disease progression.

These data indicate that POLG A1 astrocytes exhibit broad transcriptional downregulation of genes involved in neurogenesis, mitochondrial function, and synaptic support. Mitochondrial biogenesis, transport, and structural components were significantly suppressed, alongside impaired immune signaling and cytoskeletal organization. Together, these changes suggest that *POLG* mutations drive functional deterioration of A1 astrocytes, compromising their ability to support neuronal health and contributing to disease progression.

### Patient A1 astrocytes exhibited a pronounced shift toward a reactive, pro-inflammatory, and neurotoxic state

**Figure 5:**
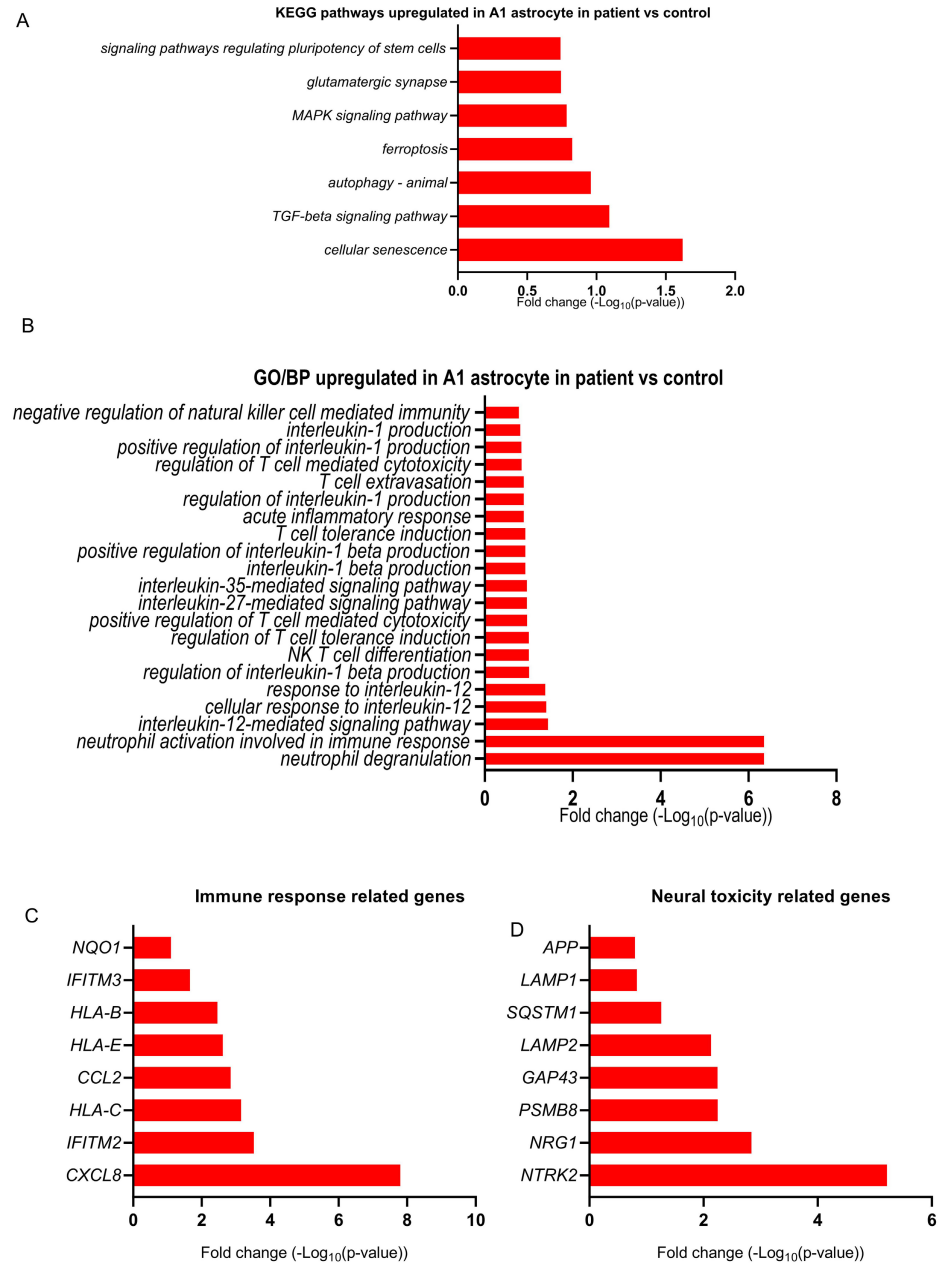
** Upregulated pathways and genes in POLG patient-derived A1 astrocytes indicate inflammatory, stress, and neurotoxic responses. (A)** KEGG pathway enrichment analysis of upregulated genes in A1 astrocytes from POLG astrocytes revealed strong activation of pathways including cellular senescence, TGF-β signaling, autophagy, ferroptosis, and MAPK signaling, indicating enhanced cellular stress, inflammation, and metabolic dysregulation. **(B)** GO BP enrichment of upregulated genes highlighted pro-inflammatory immune activation, including neutrophil degranulation, cytokine signaling (e.g., *IL-12, IL-35*), and T cell-mediated responses. **(C)** Upregulated immune-related genes in POLG A1 astrocytes, including chemokines (*CXCL8, CCL2*), MHC class I molecules (*HLA-B, HLA-C, HLA-E*), and antiviral genes (*IFITM2, IFITM3*), consistent with an immune-reactive phenotype. **(D)** Neural toxicity-related genes were also upregulated in POLG astrocytes, including markers of stress (*GAP43*), proteostasis disruption (*PSMB8, SQSTM1*), and neurodegeneration (*APP*).

To complement our GO analyses, we identified significantly enriched KEGG pathways upregulated in POLG A1 astrocytes compared to healthy controls (Figure 5A and Table S9). The KEGG pathway enrichment analysis identified significant upregulation of key pathways in patient A1 astrocytes compared to controls. Cellular senescence exhibited the highest level of enrichment, indicating increased aging-related cellular dysfunction, which may contribute to chronic inflammation in neurodegenerative conditions. The TGF-β signaling pathway was also upregulated, reinforcing its role in astrocyte reactivity and fibrotic responses. Other significantly enriched pathways included autophagy, a stress-adaptive response mechanism, and ferroptosis, an iron-dependent form of programmed cell death associated with oxidative stress and neurodegeneration. Additionally, MAPK signaling and glutamatergic synapse regulation were upregulated, suggesting altered intracellular signaling cascades and neurotransmitter homeostasis. These findings indicate a shift toward a reactive, potentially neurotoxic state in patient A1 astrocytes, characterized by increased inflammation, oxidative stress, and synaptic dysfunction.

GO BP enrichment analysis revealed significant upregulation of immune-related pathways in patient A1 astrocytes (Figure 5B and Table S10). Neutrophil degranulation was the most enriched process, suggesting a strong inflammatory response contributing to neuroinflammation. Upregulated cytokine signaling pathways, including interleukin-12-mediated and interleukin-35-mediated signaling, along with positive regulation of interleukin-1 beta production, indicate a pro-inflammatory astrocyte phenotype. Additionally, processes such as acute inflammatory response, T cell extravasation, and positive regulation of T cell-mediated cytotoxicity were enriched, reflecting increased immune system activation. Furthermore, NK T cell differentiation and response to interleukin-12 suggest broader immune system interactions, which may exacerbate neuroinflammatory conditions.

At the gene level, several immune response-related genes were significantly upregulated in patient A1 astrocytes (Figure 5C and Table S11). *CXCL8 (IL-8)* exhibited the highest log-fold change, supporting a strong pro-inflammatory response and immune cell recruitment. Additionally, *HLA-C, HLA-B, and HLA-E,* which are involved in antigen presentation and immune modulation, showed increased expression, indicating enhanced immune surveillance. *IFITM2* and *IFITM3*, associated with antiviral responses and innate immune activation, were also upregulated. Furthermore, *CCL2,* a key chemokine that facilitates monocyte and microglial recruitment, exhibited elevated expression, reinforcing the immune-activated phenotype of patient A1 astrocytes. These findings suggest that patient A1 astrocytes adopt a highly pro-inflammatory state, potentially contributing to sustained neuroinflammation and neurodegeneration.

The analysis of neural toxicity-related genes revealed significant upregulation of key factors associated with neurodegeneration and cellular stress in patient A1 astrocytes (Figure 5D and Table S12). *NTRK2*, which encodes the TrkB receptor involved in neurotrophic signaling, exhibited the highest log-fold increase, suggesting a dysregulated astrocytic stress response*. GAP43*, a marker of neuronal plasticity, and *PSMB8*, a component of the immunoproteasome linked to neuroinflammation, were also upregulated, indicating potential disruptions in synaptic remodeling and proteostasis. Moreover, lysosomal-associated proteins *LAMP2* and *LAMP1*, which regulate autophagy and cellular clearance, showed increased expression, suggesting an upregulation of degradative pathways that may be insufficient to prevent toxic protein accumulation. *SQSTM1*, a key regulator of autophagy, and *APP* (amyloid precursor protein), which is associated with Alzheimer's disease pathology, were also elevated.

To obtain a global view of pathway-level dysregulation, we performed enrichment analysis across curated biological pathways. As shown in Figure S6, ribosome and translation-related processes were among the most highly enriched, indicating increased protein synthesis or translational stress in POLG A1 astrocytes. Additional enriched pathways included spliceosome, cell cycle, and nucleocytoplasmic transport, suggesting aberrant regulation of RNA processing and nuclear-cytoplasmic signaling. Interestingly, ATP-dependent chromatin remodeling and cellular senescence were also significantly enriched, consistent with earlier findings and supporting the hypothesis of epigenetic and transcriptional reprogramming under POLG-induced stress. Enrichment of neutrophil extracellular trap formation, cytoskeletal regulation, and necroptosis points toward a reactive and damage-prone cellular state, further linking POLG A1 astrocytes to inflammatory and cytotoxic mechanisms. The inclusion of COVID-19-related signatures may reflect shared stress and immune signaling profiles rather than disease-specific responses.

To further characterize the functional reprogramming of POLG A1 astrocytes, we performed GO enrichment analysis for MF terms based on upregulated genes. As shown in Figure S7, the most enriched category was “structural constituent of ribosome”, consistent with increased ribosomal gene expression and elevated translational activity or ribosomal stress. Additional highly enriched molecular functions included mRNA 5'-UTR binding, mRNA 3'-UTR binding, and single-stranded RNA binding, which are typically involved in post-transcriptional regulation, mRNA stability, and selective translation under stress. The enrichment of unfolded protein binding, ubiquitin-like protein ligase binding, and chromatin DNA binding suggest disruptions in proteostasis, protein quality control, and epigenetic regulation, respectively.

To investigate gene regulatory dynamics in POLG A1 astrocytes, we analyzed upregulated genes for enriched GO BP terms. As shown in Figure S8, the most prominent processes included “nuclear-transcribed mRNA catabolic process, nonsense-mediated decay”, “RNA catabolic process”, and “mRNA catabolic process”, suggesting an enhancement of mRNA surveillance and degradation pathways. In parallel, multiple processes related to cotranslational protein targeting the membrane, including SRP-dependent protein targeting to ER and establishment of protein localization to ER, were enriched, alongside translational initiation. These pathways point to heightened translational machinery activity and ER engagement, possibly reflecting increased secretory demand or ER stress.

GO CC analysis of upregulated genes in POLG A1 astrocytes revealed strong enrichment in ribosomal structures, including cytosolic ribosome, ribosomal subunits, small/large ribosomal subunits, and spliceosome complex (Figure S9). These data are consistent with the observed enrichment of ribosomal function, mRNA surveillance, and translation initiation from previous KEGG and GO analyses. The presence of spliceosome-related components supports increased RNA processing activity, possibly as an adaptation to stress-induced transcriptome remodeling. Interestingly, enrichment of focal adhesion and cell-substrate junction terms suggests reorganization of cell-matrix interaction structures, which may reflect a reactive glial state and altered tissue integration.

These data indicate that POLG A1 astrocytes exhibit a reactive, pro-inflammatory phenotype characterized by upregulation of senescence, TGF-β signaling, immune activation, and stress-response pathways. Elevated expression of genes involved in autophagy, proteostasis, ribosomal activity, and chromatin remodeling suggests cellular stress and dysregulated protein homeostasis. Together, these findings highlight widespread transcriptomic reprogramming in POLG astrocytes that may drive chronic inflammation and neurotoxicity in mitochondrial disease.

### POLG astrocytes induced widespread downregulation of gene expression in DA neurons

**Figure 6:**
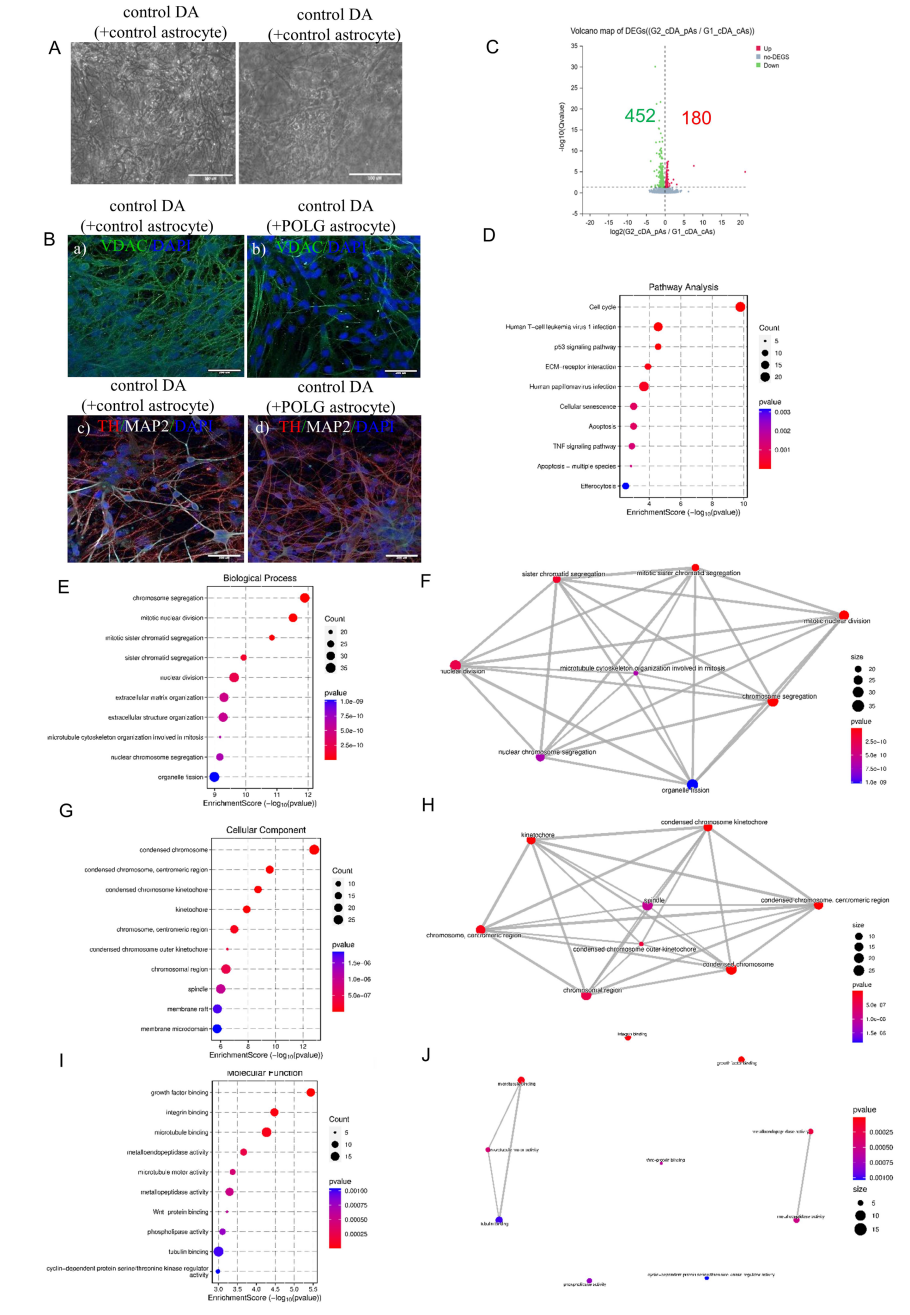
** POLG astrocytes induce transcriptional suppression and structural degeneration in co-cultured DA neurons. (A)** Brightfield images of control DA neurons co-cultured with control astrocytes (left panel) or* POLG* mutant astrocytes (right panel). POLG astrocyte co-cultures display reduced neurite complexity and signs of degeneration. The scale bar is 100 µm. **(B)** Immunofluorescence of VDAC (green), TH (red), MAP2 (white), and DAPI (blue) in control DA neurons co-cultured with control astrocyte (a, c) or POLG astrocyte co-cultures (b, d). The scale bar is 200 µm. **(C)** Volcano plot showing 632 DEGs in DA neurons co-cultured with POLG astrocytes vs DA neurons co-cultured with control astrocytes, with 180 genes upregulated and 452 downregulated. **(D)** Pathway enrichment analysis of DEGs in DA neurons co-cultured with POLG astrocytes versus control astrocyte co-cultures. Dot size represents gene count and color indicates p-value. **(E)** GO BP analysis of downregulated genes in DA neurons co-cultured with POLG astrocytes vs DA neurons co-cultured with control astrocytes shows significant repression in mitotic and cell division processes, including chromosome segregation and organelle fission. **(F)** Functional network of GO BP analysis of downregulated genes shows highlights functional interconnections among key pathways such as mitotic spindle organization, sister chromatid segregation, and chromosome condensation. **(G)** GO CC enrichment reveals downregulation of chromosomal and spindle apparatus components involved in mitosis. **(H)** Functional network of downregulated Cellular Components underscores coordinated repression of nuclear division and chromosomal structures. **(I)** GO MF terms show downregulation in microtubule and actin binding, growth factor binding, and cyclin-dependent kinase regulator activity, suggesting impaired structural integrity and signaling. **(J)** MF network shows clusters of suppressed cytoskeletal and signaling-associated protein interactions.

To investigate the functional consequences of astrocyte pathology on neuronal integrity, we performed a co-culture experiment using control DA neurons and either control or POLG astrocytes. As shown in Figure 6A, brightfield imaging revealed distinct differences in neuronal morphology after several days of co-culture. In the control (ctrl) astrocyte co-cultured with control DA condition (left), DA neurons exhibited a dense and extended neurite network with well-defined cellular bodies and active outgrowth. In contrast, when control DA neurons were cultured with POLG astrocytes (right), the neurite network appeared markedly disrupted, with fewer and thinner processes, and increased signs of neurite retraction or degeneration. To assess the impact of *POLG*-mutant astrocytes on mitochondrial health and neuronal development, we performed immunofluorescence staining for VDAC, TH, and MAP2 in control DA neurons co-cultured with either control or POLG astrocytes (Figure 6B). In cultures with control astrocytes, VDAC staining revealed a dense and organized mitochondrial network along neuronal projections, suggesting healthy mitochondrial distribution and function (Figure 6B, a). In contrast, control DA neurons co-cultured with POLG astrocytes exhibited markedly sparser VDAC signals with a punctate and fragmented pattern (Figure 6B, b), indicative of mitochondrial impairment. Consistently, co-staining for TH and MAP2 showed that DA neurons supported by control astrocytes formed robust networks with extensive neurite outgrowth and strong expression of both dopaminergic and neuronal markers (Figure 6B, c). In comparison, DA neurons co-cultured with POLG astrocytes displayed reduced neuronal density, shortened processes, and diminished branching complexity (Figure 6B, d). These findings suggest that *POLG*-mutant astrocytes exert detrimental, non-cell-autonomous effects on mitochondrial homeostasis and the development of dopaminergic neurons.

To determine the transcriptional impact of POLG astrocytes on dopaminergic neuron fate, we performed bulk RNA sequencing and compared gene expression profiles between control DA neurons co-cultured with POLG astrocytes and control DA neurons co-cultured with control astrocytes. As illustrated in the volcano plot (Figure 6C), a total of 632 genes were differentially expressed between the two groups, with 180 genes significantly upregulated (red) and 452 genes significantly downregulated (green) in the G2 group (adjusted Q < 0.05, log2 fold change > 1). The global shift in gene expression indicates that astrocyte origin significantly modulates the transcriptomic landscape of co-cultured dopaminergic neurons, consistent with morphological observations and prior pathway analyses. These DEGs were further analyzed for pathway enrichment, gene ontology categorization, and cell type-specific functional impacts.

Pathway enrichment analysis of the downregulated genes (Figure 6D) revealed significant involvement of cell cycle regulation, p53 signaling, cellular senescence, apoptosis, TNF signaling, and efferocytosis. These results suggest that neurons exposed to POLG astrocytes undergo cellular stress, cycle arrest, and activation of death pathways, consistent with a neurotoxic environment.

Further GO BP analysis of downregulated genes highlighted a strong suppression of cell division and mitotic processes in dopaminergic neurons exposed to POLG astrocytes (Figure 6E). The top enriched terms included chromosome segregation, mitotic nuclear division, sister chromatid segregation, organelle fission, and microtubule cytoskeleton organization involved in mitosis. These findings indicate a broad transcriptional repression of cell cycle-related programs, possibly reflecting cell cycle arrest, neuronal maturation block, or entry into a senescent-like state. A network representation of these GO terms (Figure 6F) revealed strong interconnections among cell cycle and mitotic regulators, suggesting a coordinated downregulation of nuclear division machinery. Notably, these biological themes align with the KEGG enrichment of p53 signaling, apoptosis, and cellular senescence, pointing to a neurodegenerative shift in transcriptional identity driven by astrocytic dysfunction.

CC enrichment analysis of downregulated genes further confirmed suppression of structures involved in chromosomal segregation and mitotic organization (Figure 6G). The most significantly enriched terms included condensed chromosome, centromeric region, kinetochore, chromosomal region, and spindle. These findings suggest an overall depletion of transcripts involved in mitotic spindle formation and chromosome condensation in neurons exposed to POLG astrocytes. A network representation (Figure 6H) revealed tight interconnectivity between these cellular components, highlighting a functionally unified downregulation of mitotic machinery. These structural annotations align with the previously identified suppression of cell cycle, organelle fission, and chromosome segregation processes at the biological process level.

MF terms among downregulated genes revealed enrichment of pathways associated with cytoskeletal regulation, signal reception, and protein activity modulation (Figure 6I). Key suppressed functions included microtubule binding, tubulin binding, microtubule motor activity, and cyclin-dependent kinase regulator activity, indicating a decline in structural maintenance and mitotic machinery engagement in neurons co-cultured with POLG astrocytes. Additionally, genes involved in growth factor binding, integrin binding, and Wnt protein binding were also downregulated, pointing to impaired extracellular signaling and reduced neuronal responsiveness to environmental cues. The network analysis (Figure 6J) further highlighted functional clustering between cytoskeletal-related binding and enzymatic activity terms, supporting the notion of coordinated suppression of neuronal structural and regulatory systems. This complements previous findings of downregulation in biological processes such as chromosome segregation and apoptotic resistance, reinforcing the hypothesis of a neurodegenerative and structurally compromised phenotype driven by pathological astrocyte signaling.

These findings suggest that POLG astrocytes impair dopaminergic neuron structure and trigger widespread transcriptional changes associated with cell cycle arrest, senescence, and neurodegeneration. Downregulated genes were enriched in pathways related to mitotic division, cytoskeletal integrity, and extracellular signaling, indicating loss of neuronal plasticity and support. Together, this points to a neurotoxic environment driven by astrocyte dysfunction that compromises neuronal survival and function.

### Astrocyte-induced transcriptional rewiring in DA neurons highlights inflammatory and synaptic remodeling responses

**Figure 7:**
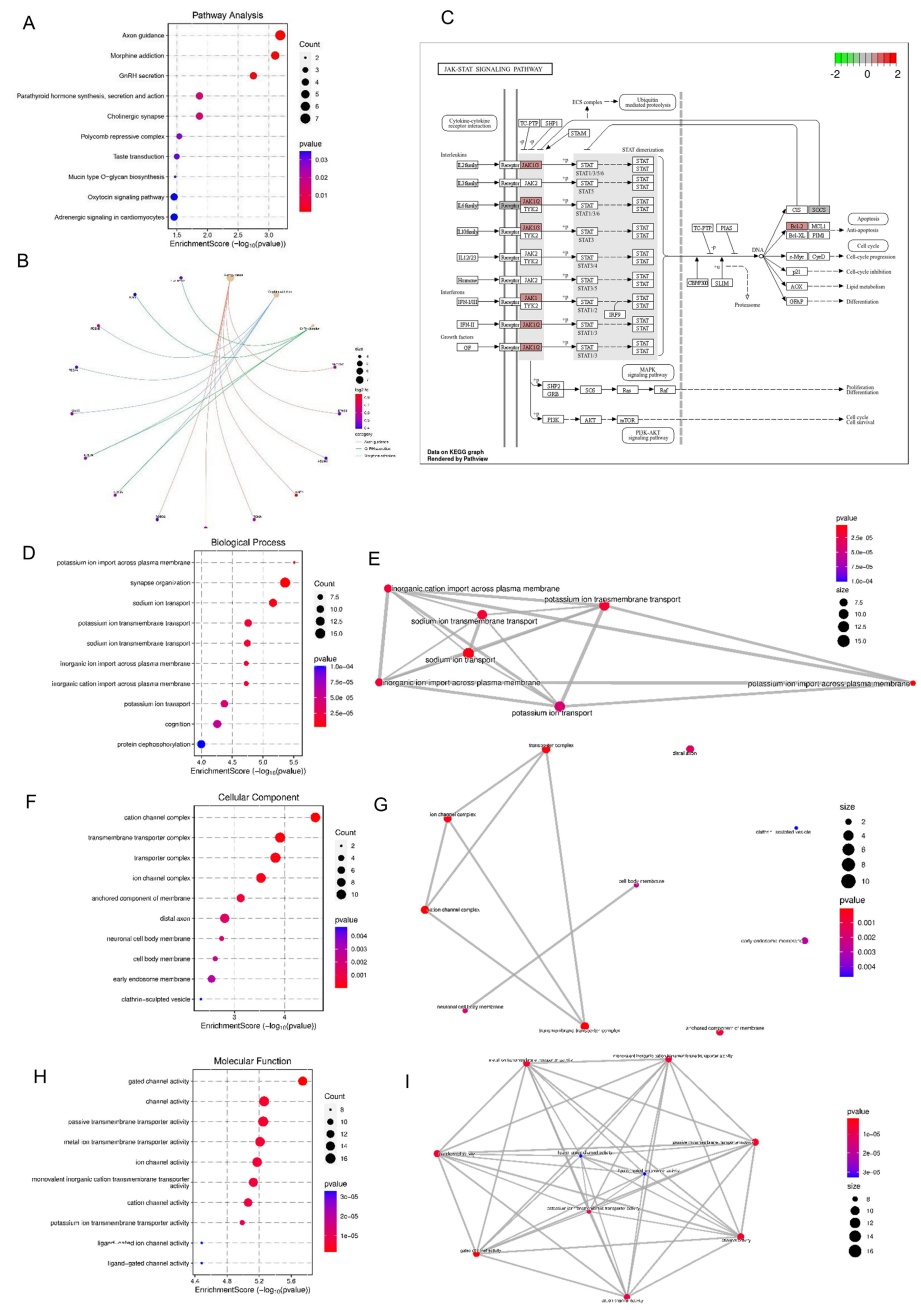
** Astrocyte-induced transcriptional reprogramming in DA neurons reveals inflammatory and excitability-related pathways. (A)** KEGG pathway enrichment analysis of upregulated genes reveals significant enrichment in pathways related to axon guidance, GnRH secretion, morphine addiction, cholinergic and oxytocin signaling, indicating neuroendocrine remodeling. **(B)** Pathway network of upregulated DEGs visualizes interconnectivity among neurodevelopmental and hormone signaling pathways. **(C)** KEGG map illustrating upregulation of the JAK-STAT signaling pathway in POLG astrocyte co-cultured DA neurons, including *IL6ST, JAK1, STAT3,* and *SOCS3*. **(D)** GO BP enrichment shows increased expression in terms related to ion homeostasis, including sodium and potassium ion transport and synapse organization. **(E)** Functional network of ion transport-related GO BP terms illustrates interconnected stress adaptation in neurons. **(F)** GO CC terms highlight enrichment in cation channel complexes, ion transporter complexes, and neuronal membrane regions, suggesting altered excitability. **(G)** Network visualization of enriched GO CC terms showing structural reorganization of transmembrane and synaptic compartments. **(H)** GO MF analysis reveals enrichment in channel activity, ion transmembrane transporter activity, and ligand-gated ion channel activity. **(I)** Network graph of enriched GO MF terms indicates coordinated activation of excitability and signal transduction components.

To further explore the functional implications of genes upregulated in DA neurons co-cultured with POLG astrocytes, we performed KEGG pathway enrichment analysis. As shown in the dot plot (Figure 7A) and pathway network (Figure 7B), the upregulated genes were significantly enriched in pathways related to neuronal development and neuroendocrine signaling. These included axon guidance, morphine addiction, GnRH secretion, cholinergic synapse, and oxytocin signaling. The most prominently enriched term, axon guidance, suggests that neurons may initiate a compensatory or stress-responsive structural remodeling program in response to POLG astrocyte-induced toxicity. Additionally, the enrichment of hormonal signaling pathways such as GnRH and oxytocin signaling may reflect altered neuromodulatory dynamics within the co-culture system.

Further pathway-specific mapping revealed significant upregulation of the JAK-STAT signaling pathway in DA neurons co-cultured with POLG astrocytes (Figure 7C). Genes encoding critical components of the JAK-STAT pathway, including *IL6ST, JAK1, STAT3,* and *SOCS3* were transcriptionally activated, suggesting an enhanced inflammatory signaling response. This is consistent with elevated levels of IL-6 observed in cytokine profiling. Activation of JAK-STAT signaling is known to promote glial reactivity, apoptotic cascades, and neuroinflammatory responses. The downstream consequences of this upregulation may include reduced neuronal resilience and increased susceptibility to damage, aligning with our earlier findings of neuronal degeneration, stress response activation, and altered mitochondrial pathways.

Gene Ontology enrichment analysis of upregulated DEGs in DA neurons co-cultured with POLG astrocytes revealed a strong association with biological processes related to ion transport and synaptic organization (Figure 7D). Specifically, we observed significant enrichment in terms such as potassium ion import across plasma membrane, sodium ion transport, synapse organization, and cognition. These changes suggest a potential compensatory response in ion homeostasis and synaptic function under astrocyte-induced stress conditions. Network visualization (Figure 7E) further confirmed the tight interconnection between ion transport-related terms, especially those involving transmembrane sodium and potassium ion dynamics, which are essential for neuronal excitability and signal transmission. Dysregulation of these pathways may reflect disturbed neurophysiological function or altered neuronal plasticity in response to pathological cues from POLG astrocytes.

Consistent with the GO-BP findings, GO-CC terms in upregulated DEGs revealed a marked enrichment of membrane-associated complexes involved in ion flux and neuronal communication (Figure 7F). The top enriched terms included cation channel complex, transmembrane transporter complex, ion channel complex, and anchored component of the membrane, indicating altered channel composition and transport structures at the neuronal membrane interface. These structures are essential for maintaining resting membrane potential and mediating action potential propagation. Network analysis (Figure 7G) showed strong interconnectivity among these components, particularly between cation/ion channel complexes and membrane anchoring domains, highlighting a potential shift in membrane-associated excitability machinery. Additional enrichment in neuronal cell body membrane, distal axon, and early endosome membrane suggests broad reorganization of subcellular compartments involved in signal transduction and vesicle trafficking, possibly reflecting adaptive changes in neurons to POLG astrocyte-derived stress signals.

These findings indicate that DA neurons co-cultured with POLG astrocytes undergo transcriptional reprogramming marked by upregulation of pathways related to axon guidance, neuroendocrine signaling, and JAK-STAT-mediated inflammation. Gene ontology analyses revealed enrichment in ion transport, synaptic organization, and membrane-associated complexes, suggesting altered excitability and compensatory structural remodeling. Together, this transcriptional shift reflects an adaptive but potentially maladaptive neuronal response to astrocyte-derived stress and inflammation.

### NR attenuates POLG astrocyte reactivity, neuroinflammation, and mitochondrial dysfunction

**Figure 8:**
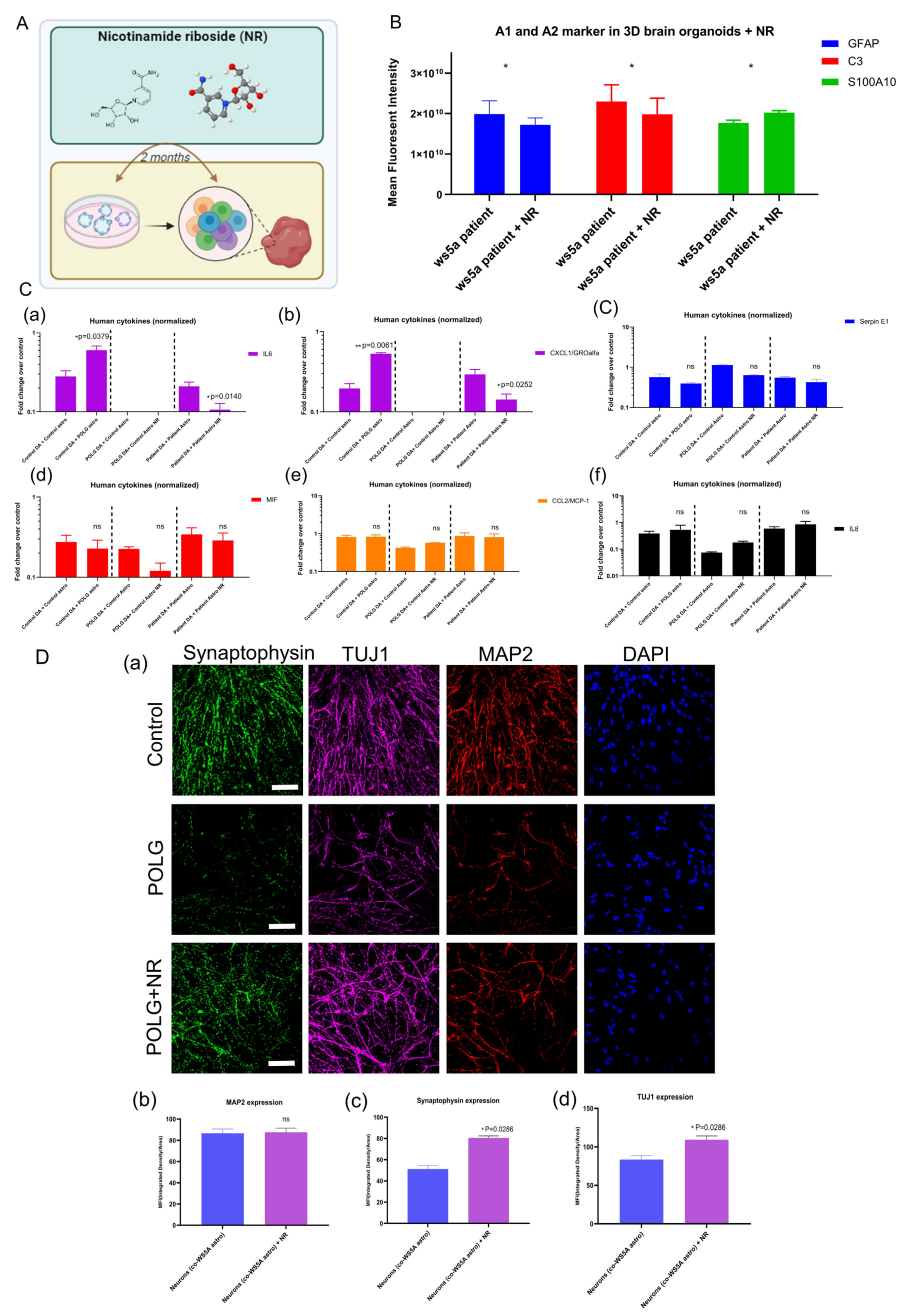
** NR alleviates POLG astrocyte reactivity, neurotoxicity, and mitochondrial dysfunction. (A)** Schematic of NR treatment in POLG cortical organoids over 2 months. **(B)** GFAP levels remain unchanged post-NR, while A1 marker C3 is reduced and A2 marker S100A10 modestly upregulated, indicating a phenotype shift toward neuroprotection. Data are presented as mean ± SEM; n = 3 biological replicates. **(C)** Cytokine profiling shows significantly reduced IL-6 and CXCL1/GROα secretion, and the levels of Serpin E1, MIF, MCP-1, and IL-8 were unaffected in NR-treated co-cultures involving POLG astrocytes and control DA neurons. Data are presented as mean ± SEM; statistical significance was determined using the Mann-Whitney U test; *p < 0.05; **p < 0.01; ns, not significant. At least three independent experiments were performed. **(D)** Representative immunofluorescence images (a) and quantification (b, c, d) of DA neurons co-cultured with astrocytes treated with NR show enhanced neurite complexity and expression of synaptic markers (Synaptophysin, MAP2, TUJ1) following NR treatment. Scale bars: 50 μm.

Building upon our previous observations of NAD⁺ metabolism dysregulation in *POLG* mutant astrocytes^30^, we evaluated the potential therapeutic impact of the NAD⁺ precursor NR on astrocyte reactivity and neurotoxicity in *POLG*-mutant astrocytes (Figure 8A). To identify an optimal dosage, POLG astrocytes were treated with a range of NR concentrations (0 mM, 0.1 mM, 0.25 mM, 0.5 mM, and 1 mM). Western blot analysis revealed that 0.5 mM NR treatment significantly reduced the expression of NESTIN, a marker of astrocyte activation, and α-SMA, a marker associated with reactive astrogliosis and scar formation (Figure S9), indicating that 0.5 mM NR can effectively suppress astrocytic reactivity.

To further characterize these effects, we assessed the expression of canonical astrocyte markers following two months of 0.5 mM NR treatment in POLG brain organoids. Immunofluorescence quantification demonstrated that GFAP levels remained unchanged, suggesting that the total astrocyte population was not significantly affected (Figure 8B). However, NR-treated POLG organoids showed reduced expression of the A1-type reactive astrocyte marker C3, along with a modest upregulation of the A2-type neuroprotective marker S100A10. This finding suggests that NR induces a phenotypic shift in astrocytes from a neurotoxic A1 state toward a more homeostatic or neuroprotective A2 profile.

To address dose optimization and potential toxicity, we performed a dose-response study in POLG patient-derived astrocytes. NR increased mitochondrial membrane potential (TMRE normalized to mitochondrial mass) with a maximal effect at 0.5 mM, whereas higher doses (1-2 mM) showed reduced benefit (Figure S11a). Importantly, an MTT viability assay demonstrated decreased viability in POLG astrocytes at ≥1 mM NR, indicating dose-limiting toxicity at higher concentrations (Figure S11b). Therefore, we define 0.5 mM as the “optimal” dose because it provides the strongest functional rescue while maintaining viability.

Next, we evaluated whether NR could modulate the inflammatory secretome of POLG astrocytes in dopaminergic neuron co-cultures. Human cytokine array profiling, which detecting 36 human cytokines, revealed that baseline secretion of 8 inflammatory mediators, including CCL2/MIP-1, CXCL1/GROα, IL-6, IL-8, MIF, Serpin E1, MIP-1α, CCL5, were detectable and comparable between POLG astrocytes and control astrocytes (Figure S12). However, IL-6 and CXCL1/GROα secretion was significantly elevated when POLG astrocytes were co-cultured with control DA neurons (Figure 8C, panels a-b) but absent in co-cultures of POLG DA neurons with control astrocytes, implicating POLG astrocytes as the source of these cytokines. When treating with NR, the cytokine array profiling showed that baseline secretion of six mediators, including CCL2/MIP-1, CXCL1/GROα, IL6, IL8, MIF, Serpin E1, was broadly comparable between control and POLG astrocyte co-cultures (Figure S13). Importantly, following treatment with 0.5 mM NR, both IL-6 and CXCL1/GROα levels were markedly reduced, supporting a NR-mediated suppression of astrocyte-derived pro-inflammatory signaling (Figure 8C, panels a-b).

To assess the impact of NR on neuronal health, we conducted 20-day co-culture experiments using POLG or control astrocytes with or without NR. Brightfield imaging showed that NR treatment improved neuronal network complexity, with increased neurite outgrowth in co-cultures involving either POLG DA neurons + control astrocytes or POLG DA neurons + POLG astrocytes (Figure S14). Immunostaining further confirmed enhanced expression of neuronal maturation and synaptic markers, including synaptophysin, βIII-tubulin (TuJ1), and MAP2, upon NR treatment (Figure 8D), indicating functional rescue of POLG neuronal morphology.

Collectively, these results demonstrate that NR treatment attenuates astrocyte reactivity, reduces pro-inflammatory cytokine output, improves neuronal morphology, and partially restores mitochondrial function in POLG-derived models. This highlights NR as a promising metabolic modulator for mitigating astrocyte-mediated neurotoxicity in mitochondrial encephalopathies.

### NR enhances mitochondrial bioenergetics via a PGC-1α-complex I axis in POLG astrocytes

**Figure 9:**
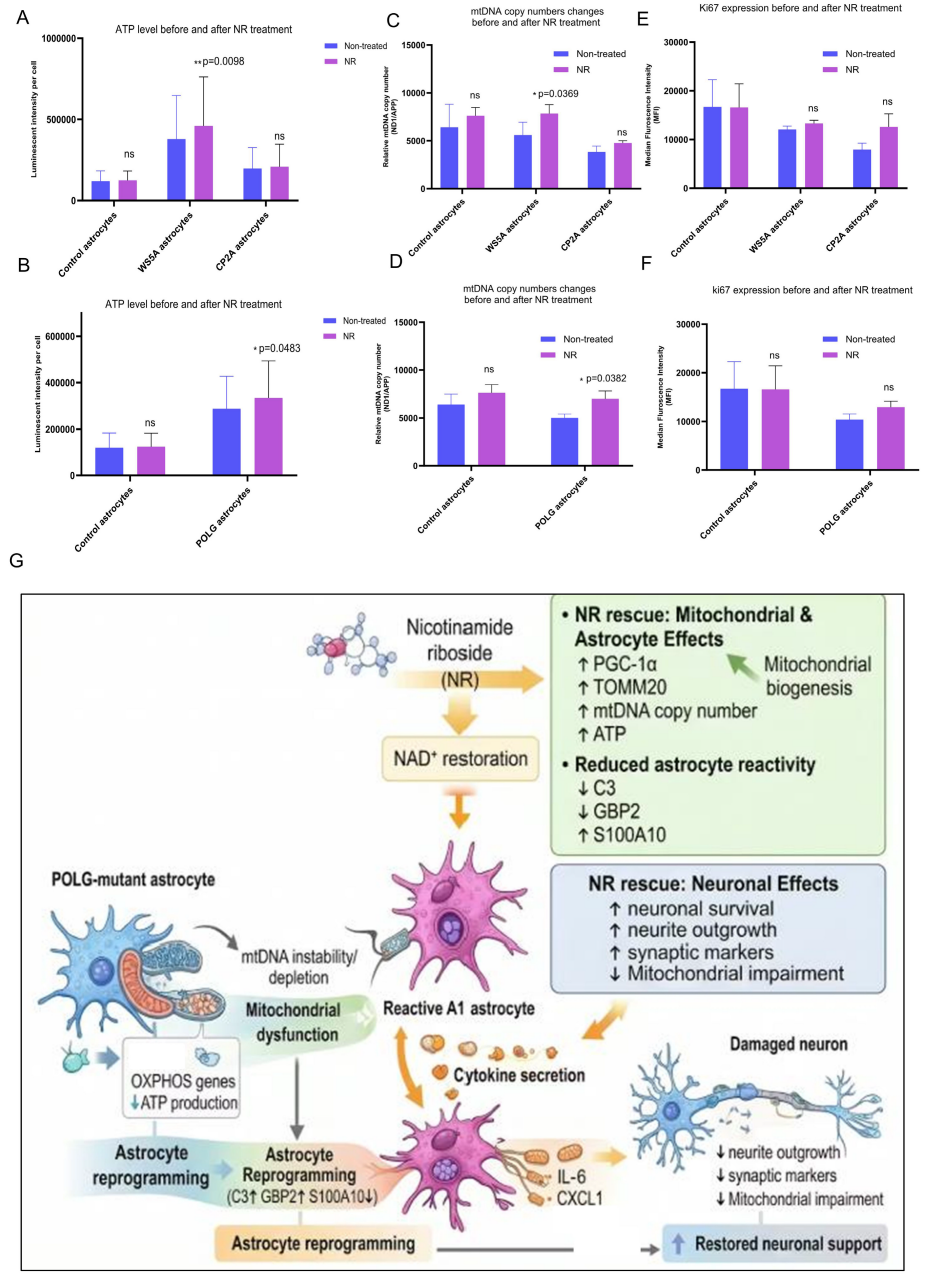
** NR enhances mitochondrial bioenergetics in POLG astrocytes. (A-B)** Intracellular ATP levels were quantified in two POLG patient-derived astrocyte lines (CP2A and WS5A) and in isogenic controls before and after NR treatment. NR increased ATP production in both POLG lines and combined lines. **(C-D)** mtDNA copy number was elevated following NR treatment in WS5A and CP2A lines and pooled data in NR treated astrocytes compared with untreated POLG astrocytes. (**E-F**) Ki67 expression showed a mild, nonsignificant increase after NR treatment in both patient lines. **(G)** Schematic model illustrating how *POLG* mutations drive astrocyte-mediated neuronal dysfunction and how NR treatment mitigates these effects. Data in A-F are presented as mean ± SEM; Statistical significance was determined using a two-way mixed-effects model (REML); *p < 0.05; **p < 0.01; ns, not significant. At least three independent experiments were performed.

To test whether NR rescues mitochondrial dysfunction in POLG astrocytes, we quantified intracellular ATP, mtDNA copy number, and Ki67. NR increased ATP in both patient lines, with a modest rise in CP2A and a more pronounced elevation in WS5A astrocytes that approached isogenic-control levels (Figure 9A). When pooled, the two lines showed a significant overall ATP increase, indicating that NR improves cellular energy production across POLG genotypes (Figure 9B).

mtDNA copy number also rose after NR treatment, clearly in WS5A and modestly but consistently in CP2A (Figure 9C). Combined analysis confirmed a significant mtDNA elevation relative to untreated POLG astrocytes (Figure 9D), consistent with enhanced mitochondrial biogenesis or stabilization of mtDNA maintenance.

By contrast, Ki67 showed only a small, nonsignificant increase in both lines (Figure 9E), and the pooled data indicated no material change in proliferation (Figure 9F), suggesting that NR's primary benefit is bioenergetic rather than mitogenic.

To explore potential mitochondrial mechanisms underlying NR's protective effects, we performed Western blotting for mitochondrial biogenesis and respiratory markers. We observed a significant increase in PGC-1α, a master regulator of mitochondrial biogenesis, along with elevated levels of Complex I (NDUFB10) in NR-treated POLG astrocytes (Figure 9G). However, levels of Complex IV (COXIV) and the mitochondrial outer membrane protein TOMM20 remained unchanged, suggesting that NR selectively enhances mitochondrial biogenesis and Complex I activity, but does not broadly affect all mitochondrial compartments or respiratory chain complexes.

In summary, these data demonstrate that NR supplementation enhances mitochondrial bioenergetics in POLG astrocytes, elevating ATP production and mtDNA copy number, particularly in the WS5A line, without affecting proliferation, and acts through a selective PGC-1α-complex I-dependent pathway.

## Discussion

In this study, we investigated how *POLG* mutations influence astrocyte reactivity, neuronal health, and disease-associated cellular programs. Building on our previous findings that POLG mutant astrocytes adopt an A1-like neurotoxic phenotype in 2D models^25^, we demonstrated here that this phenotype is robustly recapitulated in 3D brain organoids. Importantly, single-cell RNA sequencing revealed a profound skewing of astrocyte differentiation toward the A1 subtype, accompanied by a depletion of neuroprotective A2 astrocytes and neuronal populations. This shift in glial composition likely contributes to a non-cell autonomous mechanism of neuronal vulnerability in POLG-associated mitochondrial disease.

The marked expansion of A1 astrocytes in POLG organoids mirrors previous reports in neuroinflammatory and neurodegenerative contexts, such as Alzheimer's disease, multiple sclerosis and Parkinson's disease^12^. However, unlike these models where A1 induction is primarily driven by external immune insults or aging, our findings suggest that mitochondrial DNA maintenance defects alone are sufficient to drive intrinsic transcriptional reprogramming toward a neurotoxic astrocyte identity. This is evidenced by elevated expression of canonical A1 genes such as *CXCL8, SERPINE1, GAP43*, and *CCN2*, as well as significant activation of TGF-β signaling, autophagy, and oxidative stress pathways, hallmarks of astrocyte reactivity and senescence.

Mitochondrial dysfunction has long been implicated in POLG-related pathology, with most studies focusing on neuronal or systemic effects. Here, we demonstrate that POLG astrocytes exhibit extensive downregulation of oxidative phosphorylation, mitochondrial transport and import machinery, and structural mitochondrial components. These transcriptomic alterations suggest a collapse of mitochondrial homeostasis in A1 astrocytes, potentially contributing to chronic inflammatory signaling, impaired metabolic support for neurons, and an inability to buffer extracellular stress. Notably, this aligns with previous work showing that *POLG* mutations impair mitophagy and mitochondrial quality control in neurons^29^,^32^, raising the possibility that astrocytes may also contribute to metabolic insufficiency in the neuro-glial network.

Importantly, the downregulation of mitochondrial genes in POLG A1 astrocytes aligns with our previous findings and reinforces the notion that astrocytes^25^, like neurons, are highly susceptible to mtDNA instability and bioenergetic failure. Unlike neurons, however, astrocytes have traditionally been considered metabolically resilient. Our results challenge this assumption and provide direct evidence that mitochondrial dysfunction in astrocytes can profoundly impact their homeostatic and neurosupportive roles, particularly under disease-associated stress. This echoes findings from murine models of mitochondrial encephalopathy, where astrocyte-specific respiratory chain dysfunction led to spontaneous epileptic seizures and neuroinflammation^13^.

Another key observation is the widespread transcriptional suppression of genes involved in cytoskeletal structure, postsynaptic interaction, and synaptic scaffolding. This likely impairs tripartite synapse formation, a crucial aspect of astrocyte-mediated neurotransmitter regulation and neuronal plasticity. Prior studies in models of Alzheimer's and ALS have shown that reactive astrocytes lose their ability to buffer glutamate, regulate synaptic clefts, and maintain perisynaptic homeostasis, leading to excitotoxicity and neuronal damage^33^,^34^. Our findings in POLG models extend this concept by showing that intrinsic mitochondrial damage can recapitulate these phenotypes, suggesting a mitochondria-cytoskeleton-synapse axis that may be a shared vulnerability in multiple neurodegenerative conditions.

From a mechanistic perspective, the transcriptional repression of oxidative phosphorylation genes and the downregulation of key mitochondrial components such as the TIM23 import system suggests impaired mitochondrial protein import and turnover. This dysfunction may promote the accumulation of damaged mitochondria and increased reactive oxygen species (ROS) production, thereby exacerbating inflammation and metabolic stress. Interestingly, although several autophagy-related genes, including *BECN1* and *LC3B*, were transcriptionally upregulated in POLG astrocytes, our DALGreen assay revealed reduced basal autolysosomal activity. This discrepancy likely reflects a compensatory transcriptional response to impaired autophagic flux. Mitochondrial dysfunction is known to activate stress-responsive transcriptional programs that enhance the expression of autophagy and lysosomal genes in an attempt to restore mitochondrial quality control^35^,^36^. However, defects in downstream autophagy-lysosome processes, such as impaired lysosomal acidification or inefficient autophagosome degradation, may prevent completion of autophagic flux. Such discordance between transcriptional activation and impaired functional autophagy has been reported in several neurodegenerative and mitochondrial disease models^37^,^38^.

The observed transcriptional repression in DA neurons co-cultured with POLG astrocytes underscores the profound impact of glial dysfunction on neuronal identity and survival. Suppression of cell cycle genes, such as those involved in mitotic spindle formation and chromosome segregation, reflects a state of irreversible cell cycle arrest, possibly associated with a senescence-like phenotype in post-mitotic neurons. This echoes findings from studies of aging and neurodegeneration, where aberrant re-entry into the cell cycle has been recognized as a hallmark of neuronal vulnerability39. The concurrent upregulation of p53 and apoptosis pathways supports this notion and indicates that astrocyte-derived stress signals may push neurons toward a path of programmed cell death.

The strong enrichment of pathways related to cytoskeletal disassembly, reduced Wnt signaling, and impaired integrin/growth factor binding suggests a breakdown in structural homeostasis and intercellular communication. These changes are consistent with disrupted astrocyte-neuron crosstalk and loss of trophic support. Previous work has demonstrated that astrocytes regulate synapse formation, pruning, and plasticity through both contact-mediated interactions and secreted factors^40^. In the context of POLG astrocyte dysfunction, our findings reveal that this supportive role is compromised, leading to dendritic fragmentation and reduced neurite outgrowth, as evidenced by reduced MAP2 and βIII-tubulin signals. The decrease in growth factor-responsive genes further supports a model in which POLG astrocytes fail to provide adequate neurotrophic signaling, potentially contributing to impaired synaptic maintenance and axonal transport.

Importantly, the upregulation of JAK-STAT signaling and cytokine-associated genes in DA neurons suggests that POLG astrocytes impose a pro-inflammatory environment that neurons can sense and respond to transcriptionally. Elevated STAT3 signaling, which we observed in co-cultured neurons, has been implicated in both neuronal injury responses and degenerative outcomes, depending on context^41^,^42^. This raises the possibility that neuronal stress responses in POLG disease may not be entirely cell autonomous but instead driven by astrocyte-derived factors such as IL-6, CXCL1/GROα, or other secreted mediators.

These findings support a broader model of non-cell-autonomous neurodegeneration in mitochondrial diseases. While *POLG* mutations have been extensively studied in neurons, our study highlights the critical role of astrocytes in modulating the neuronal environment. This is consistent with emerging paradigms in ALS, Alzheimer's disease, and Parkinson's disease, where glial dysfunction has been implicated in disease onset and progression^12^,^43^. In particular, our work suggests that astrocyte mitochondrial dysfunction alone is sufficient to drive widespread transcriptional reprogramming in neighboring neurons, even in the absence of neuronal *POLG* mutations. This raises the possibility that correcting astrocytic pathology could rescue neuronal function, a hypothesis supported by recent work using NAD⁺ supplementation and metabolic reprogramming^44^.

Furthermore, the functional impairment of ion transport and synaptic organization pathways in neurons indicates that astrocytic pathology leads to early, pre-degenerative changes that may not immediately involve cell death but instead contribute to gradual loss of network function. This opens an opportunity for therapeutic intervention at early stages of disease progression before irreversible neurodegeneration sets in.

The ability of NR to mitigate POLG astrocyte-induced pathology in our model supports the growing recognition of NAD⁺ precursors as therapeutic candidates for mitochondrial and neurodegenerative diseases. Prior studies have demonstrated that NAD⁺ depletion is a hallmark of mitochondrial dysfunction and cellular aging, and that replenishment via NR or nicotinamide mononucleotide (NMN) can rescue mitochondrial respiration, reduce oxidative stress, and promote cellular survival across multiple systems^44^,^45^. In the context of POLG disease, where impaired oxidative phosphorylation and mitochondrial DNA maintenance are central features, our findings extend these insights by highlighting NR's efficacy in reducing astrocytic reactivity.

Mechanistically, NR appears to act on multiple pathological axes in POLG astrocytes. First, the observed reduction in NESTIN suggests attenuation of reactive astrogliosis, which is typically associated with scar formation and restricted regenerative capacity. The accompanying decrease in C3 and increase in S100A10 expression indicates a phenotypic rebalancing from a neurotoxic A1 state toward a more supportive A2 phenotype. This shift has functional implications, as A1 astrocytes have been shown to promote neuronal death, while A2 astrocytes secrete neurotrophic factors and facilitate synaptic repair^12^.

In addition to modulating astrocyte identity, NR treatment led to a reduction in IL-6 and CXCL1/GROα secretion-two key pro-inflammatory cytokines implicated in neuroinflammation and blood-brain barrier breakdown. This aligns with previous findings where NR was shown to suppress NF-κB activation and dampen the pro-inflammatory secretome in models of neurodegeneration^46^. Given the upregulation of JAK-STAT signaling in DA neurons co-cultured with POLG astrocytes, the ability of NR to suppress upstream cytokine output may help break this deleterious feedback loop, preserving neuronal resilience.

These results add to a growing body of literature supporting metabolic reprogramming of glial cells as a viable strategy for treating mitochondrial encephalopathies and neurodegenerative diseases. Notably, NR has already entered clinical testing in conditions such as Parkinson's disease and mitochondrial myopathy^47^,^48^, providing translational momentum for its application in POLG syndromes. However, the extent to which NR-mediated benefits depend on astrocyte-specific effects versus systemic NAD⁺ replenishment remains to be fully elucidated.

Our findings, while derived from *in vitro* iPSC-based models, closely align with clinical manifestations observed in POLG-related mitochondrial encephalopathies, particularly Alpers-Huttenlocher syndrome and POLG-associated epilepsy, where neuroinflammation, progressive neuronal loss, and astrocytic dysfunction are prominent pathological hallmarks. The observed expansion of A1 neurotoxic astrocytes and concomitant neuronal vulnerability in our models provide a mechanistic framework that may underlie these disease phenotypes. Thus, our data establishes a direct link between *POLG* mutation-induced astrocytic reactivity, and the neurodegenerative features observed in defined POLG-related disorders.

### Limitations

A limitation of this study is that our findings are restricted to iPSC-derived organoid and co-culture models, without *in vivo* validation in *POLG*-mutant animals. While these systems are valuable for dissecting cell-intrinsic and astrocyte-neuron interactions, they cannot fully capture the complexity of an intact organism. Notably, *in vivo* studies in mitochondrial disease models have already provided proof-of-concept for NR's therapeutic effects. In a SCO2 knockout/knockin mouse model, NR supplementation increased NAD⁺ levels, activated SIRT1, enhanced oxidative phosphorylation gene expression, improved respiratory chain activity, and corrected motor deficits^49^. These results support the translational relevance of our *in vitro* findings and suggest that targeting NAD⁺ metabolism and Sirt1 activation may be a viable therapeutic strategy for POLG-related disorders. Future studies will extend our work into *POLG*-mutant animal models to evaluate whether the observed reduction in astrocyte reactivity and improvement in neuronal function can be recapitulated *in vivo*.

Another limitation is that most analyses were performed on organoids on day 30, which, although displaying neural stratification, may not fully recapitulate the complexity of mature neuronal networks or the terminal differentiation of astrocytes observed *in vivo*. To address functional maturation, we examined the expression of synaptic markers (synaptophysin), which confirmed the presence of developing synaptic structures^28^. However, electrophysiological analyses would provide a more comprehensive assessment of neuronal activity and network functionality. Due to equipment limitations, these assays were not performed in the current work, but future studies will include long-term organoid cultures (e.g., ≥ 90 days) and electrophysiological recordings to more fully evaluate neuronal and astrocytic maturation.

A further limitation is that our scRNA-seq analysis was restricted to astrocytes and neurons. While this enabled detailed investigation of astrocyte reprogramming and astrocyte-neuron interactions, it did not include microglia or other immune cell types that play essential roles in shaping the inflammatory microenvironment. As microglia-astrocyte crosstalk is a central driver of neuroinflammation, the lack of microglial representation may underestimate the contribution of intercellular immune interactions. Future studies will therefore extend the scope of single-cell analyses to include microglia and other glial/immune cell types, either through co-culture models or immune-enriched organoid systems, to capture the synergistic effects of multiple cell populations in POLG-related pathology.

In addition, our classification of astrocytes into A1 and A2 subtypes was based on classical markers such as C3 and S100A10. While this framework is widely used, emerging evidence indicates that astrocyte reactivity is not strictly binary but rather exists along a continuum of phenotypes, including intermediate and context-specific states. Our analysis may therefore underrepresent the full spectrum of astrocytic diversity. Future studies will incorporate single-cell trajectory analyses, such as Monocle or RNA velocity, to capture the dynamic process of astrocyte state transitions and provide a more comprehensive understanding of reactive astrocyte heterogeneity in POLG-related pathology.

Although our analyses of neuronal health combined morphological characterization with transcriptomic profiling and synaptic marker expression, we did not directly assess neuronal functionality. Electrophysiological recordings would provide a more comprehensive measure of neuronal network activity, while behavioral studies in *POLG*-mutant animals would further validate the functional impact of *POLG* mutations and NR treatment at the organismal level. These assays were not feasible within the scope of the present work, but future studies will integrate *in vitro* electrophysiological analyses and *in vivo* behavioral testing to strengthen the functional interpretation of our findings.

Finally, while we showed that NR reduces astrocyte reactivity and improves neuronal health, our study does not fully establish whether these effects are astrocyte-specific or whether they extend to other cell types or systemic mechanisms. Given that NAD⁺ metabolism can influence neurons, microglia, oligodendrocytes, and vascular endothelial cells, NR may exert broader effects within the neurovascular unit. Future studies using cell-type-specific genetic models, vascularized or immune-enriched organoids, and single-cell multi-omics analyses will be essential to dissect the cell-specific versus systemic actions of NR in POLG-related pathology.

Although mtDNA mutation load was not directly assessed by deep sequencing in this study, the observed increase in mtDNA content, together with elevated ATP levels, indicates that NR improves mitochondrial replication and energy production in POLG astrocytes. The high-depth sequencing of mtDNA would provide valuable insight into mutation heterogeneity and stability, and we plan to address this in future work.

### Future perspectives

Building on our findings and prior work^29^, we propose a mechanistic framework in which POLG-driven mtDNA and respiratory chain defects impair mitophagy and disrupt NAD⁺-sensitive signaling (SIRT/AMPK-mTOR axis) in astrocytes, biasing them toward neurotoxic A1-like states with elevated cytokines (e.g., IL-6, GROα) and complement, which in turn compromise neuronal structure and viability. NR restores NAD⁺-dependent control over these programs, normalizes organelle quality control, and reduces astrocyte-mediated toxicity. Future studies will aim to deepen mechanistic insights by mapping signaling crosstalk within mitophagy pathways, identifying protein-protein interactions that coordinate mitochondrial stress responses, and exploring epigenetic regulation of astrocyte phenotypes through chromatin accessibility and histone modifications. Applying trajectory inference and multi-omics approaches will further capture intermediate astrocyte states and their dynamic transitions. Such work will provide a more comprehensive view of how *POLG* mutations rewire cellular pathways to drive neurodegeneration and may highlight novel therapeutic targets for POLG-related mitochondrial encephalopathies.

In this study, we established a human iPSC-derived *in vitro* model to investigate how *POLG* mutations alter astrocyte function and influence neuronal health. Single-cell transcriptomic analysis revealed that *POLG* mutations drive a shift in astrocyte identity toward a reactive A1-like phenotype characterized by increased inflammatory gene expression and impaired mitochondrial and neuro-supportive functions. POLG astrocytes exhibited elevated secretion of inflammatory cytokines, including IL-6 and GROα, together with transcriptional signatures associated with mitochondrial dysfunction, cellular stress, and senescence. Importantly, astrocyte-neuron co-culture experiments demonstrated that POLG astrocytes exert neurotoxic effects on dopaminergic neurons, leading to impaired neuronal morphology and widespread transcriptional repression of pathways related to cell cycle regulation, cytoskeletal organization, and neuronal excitability.

Treatment with the NAD⁺ precursor nicotinamide riboside (NR) partially reversed these pathological changes. NR reduced astrocyte reactivity, modulated A1/A2 marker expression, decreased pro-inflammatory cytokine secretion, and improved neuronal morphology and synaptic marker expression in the astrocyte-neuron co-culture system. In addition, NR enhanced mitochondrial function in POLG astrocytes, as indicated by increased ATP production and elevated mtDNA copy number. Consistent with our previous study^29^, NR also promotes mitochondrial biogenesis through upregulation of regulators such as PGC-1α and mitochondrial respiratory chain components including Complex I.

To integrate these findings, we propose a model summarizing the mechanism of astrocyte-mediated neurotoxicity in POLG disease (Figure 9G). In this model, *POLG* mutations disrupt mtDNA maintenance and oxidative phosphorylation in astrocytes, leading to mitochondrial dysfunction and metabolic stress. These changes promote the transition of astrocytes toward a reactive A1-like state, characterized by increased expression of inflammatory markers such as C3 and GBP2 and reduced expression of the neuroprotective marker S100A10. Reactive astrocytes subsequently release inflammatory cytokines, including IL-6 and CXCL1, which impair neuronal integrity and contribute to reduced neurite outgrowth, decreased synaptic marker expression, and mitochondrial dysfunction in neurons. NR-mediated NAD⁺ augmentation restores mitochondrial homeostasis, attenuates astrocyte inflammatory activation, and improves astrocyte-mediated neuronal support.

Together, our findings identify astrocyte mitochondrial dysfunction as a key driver of POLG-associated neurodegeneration and highlight NAD⁺ augmentation as a promising therapeutic strategy for POLG-related mitochondrial encephalopathies.

## Supplementary Material

Supplementary figures and tables.

## Data Availability

All other data is available from the corresponding author upon request.
