# Supplementary material for: Nicotinamide riboside reduces glial inflammation and boosts mitochondrial function
Source: Int J Biol Sci. 2026 Jun 4;22(11):6035–63. doi: 10.7150/ijbs.119262 (PMC13282778; doi:10.7150/ijbs.119262)
Supplement: Supplementary file 1 — Supplementary figures and tables. [file ijbsv22p6035s1.pdf]

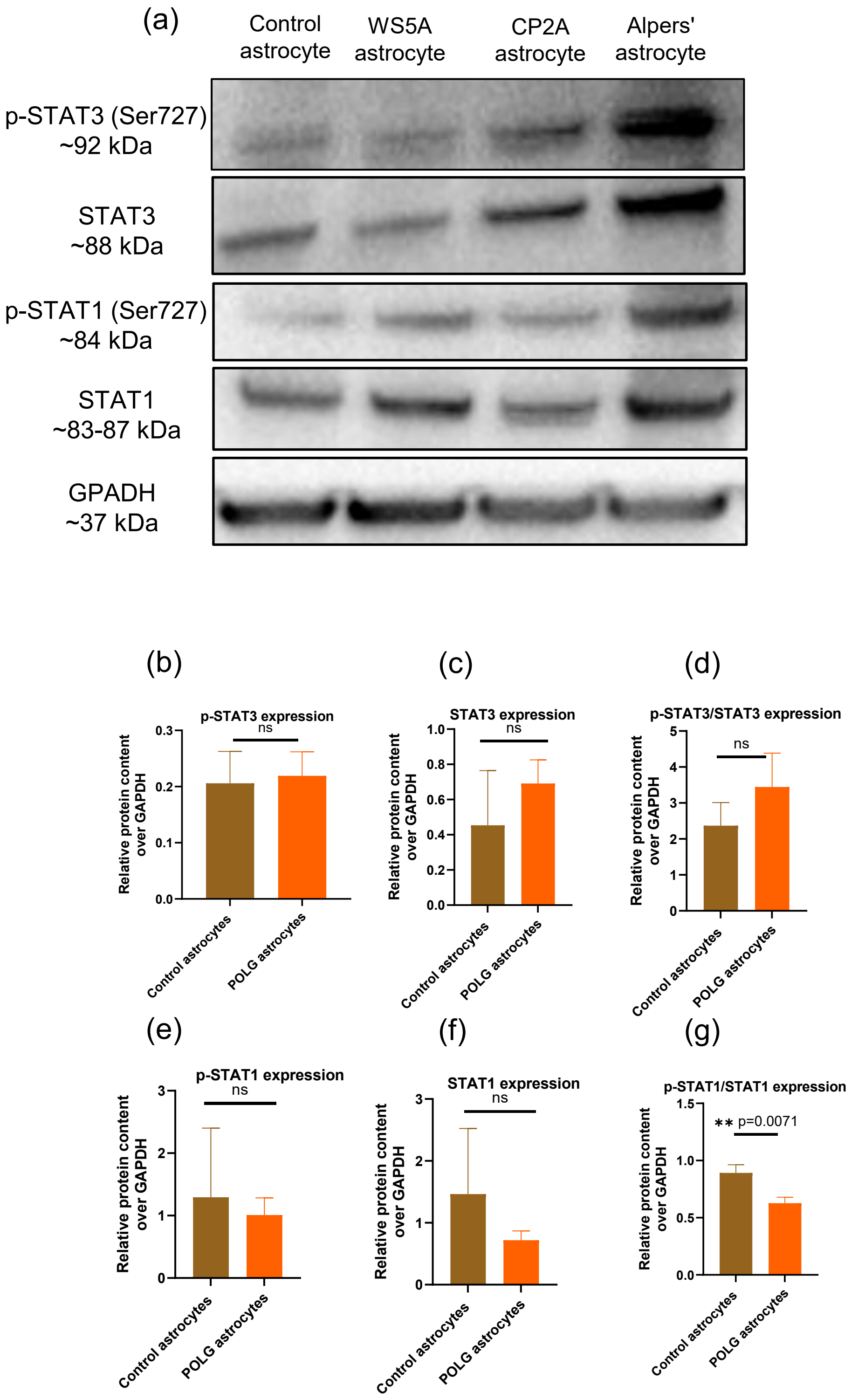

Figure S1. Western blot analysis of STAT3 and STAT1 signaling pathways.

(a) Representative Western blot analysis of p-STAT3 (Ser727), STAT3, p-STAT1 (Ser727), and STAT1, with GAPDH used as a loading control. (b, c, e, f) Quantification of relative protein expression levels of p-STAT3 (Ser727), STAT3, p-STAT1 (Ser727), and STAT1, normalized to GAPDH. (d, g) Quantification of phosphorylation levels represented as p-STAT3 (Ser727)/STAT3 and p-STAT1 (Ser727)/STAT1 ratios. Densitometric analysis of Western blot bands was performed using ImageJ software. Data are presented as mean ± SEM from independent biological replicates. Statistical significance was determined using the Mann–Whitney U test. $^{**}p < 0.01$; ns, not significant. POLG astrocytes were derived from three independent patient lines: WS5A (p.W748S/W748S), CP2A (p.A467T/W748S), and an Alpers' syndrome patient carrying compound heterozygous mutations A467T (c.1399G>A) and P589L (c.1766C>T).

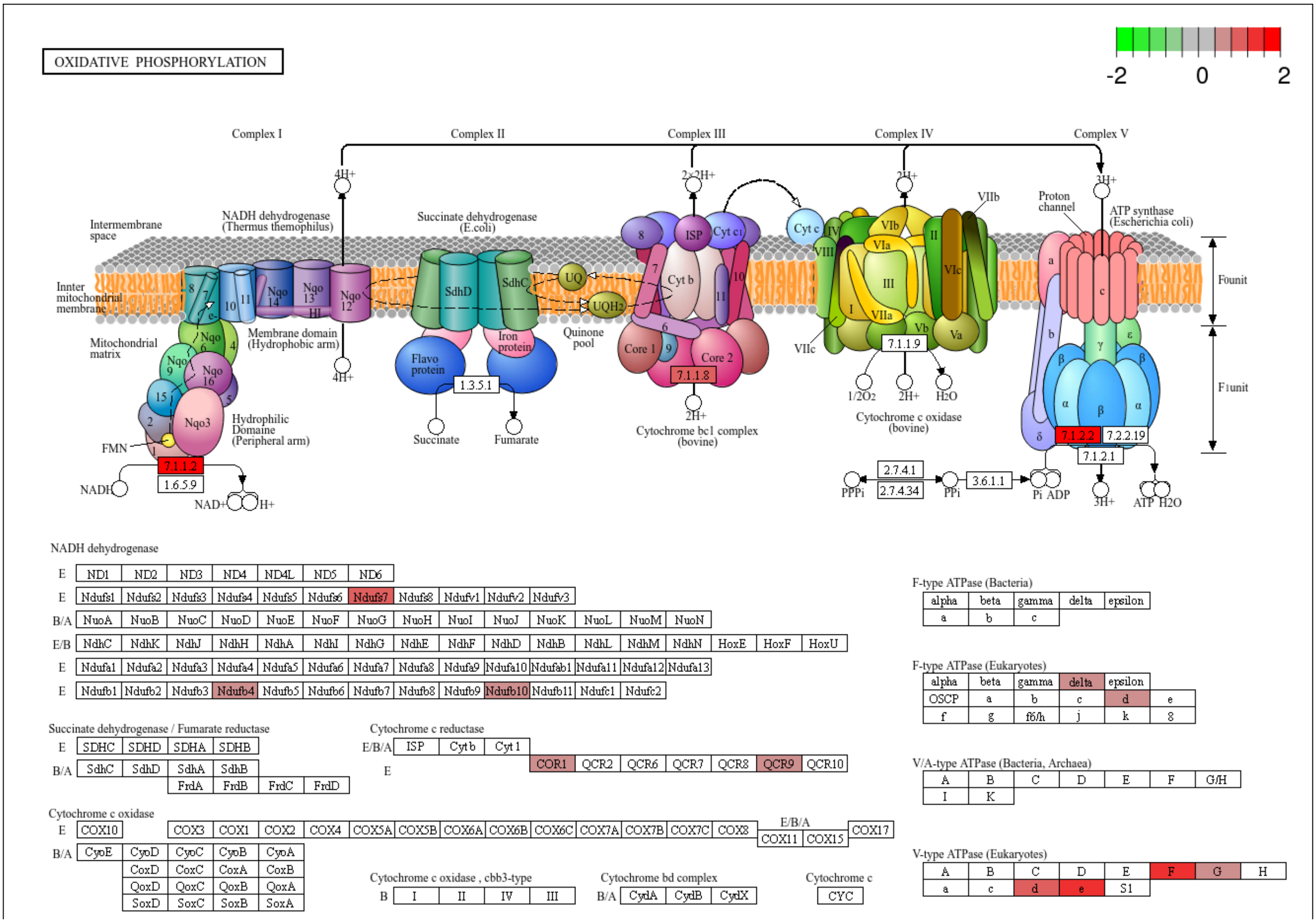

Figure S2. KEGG pathway mapping of oxidative phosphorylation reveals downregulation of mitochondrial respiratory complexes in POLG A1 astrocytes.

KEGG pathway diagram of the oxidative phosphorylation cascade shows differential expression of genes in POLG A1 astrocytes compared to isogenic controls. Color coding indicates gene expression changes, with red representing downregulation and green representing upregulation (scale bar: log2 fold change from –2 to 2). Multiple subunits of Complex I (NADH dehydrogenase; e.g., *NDUFB4, NDUFS5, NDUFA4*), Complex IV (cytochrome c oxidase; e.g., *COX6C*), and Complex V (ATP synthase; e.g., *ATP5E, ATP5G2, ATP5J2*) were notably downregulated, indicating impaired mitochondrial electron transport chain function and ATP production. This supports the hypothesis of mitochondrial respiratory dysfunction in POLG astrocytes, consistent with observed phenotypes of metabolic stress and reduced neuro-supportive capacity.

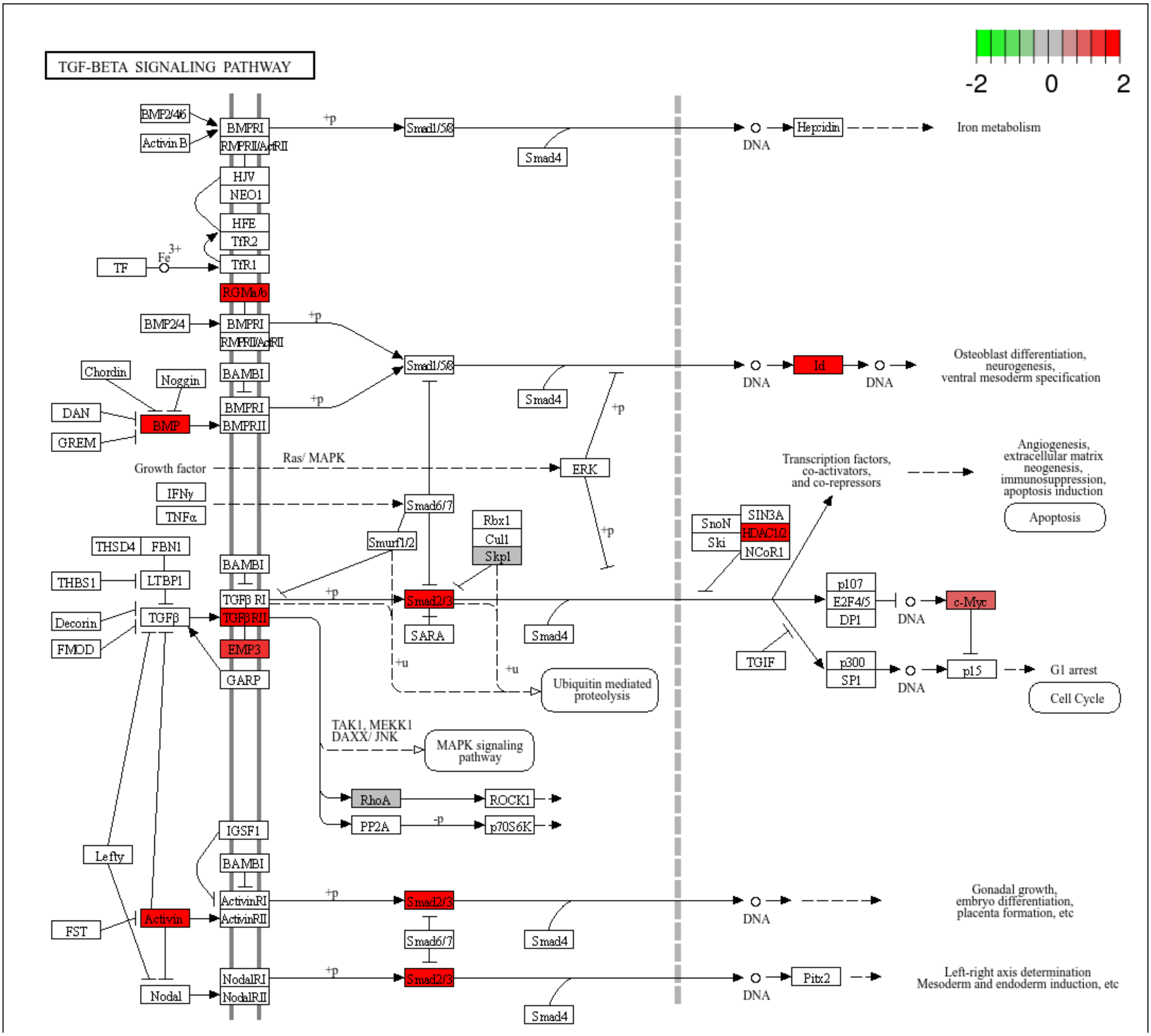

Figure S3. KEGG pathway analysis reveals upregulation of TGF-β signaling in POLG A1 astrocytes.

Pathway map of the TGF-β signaling cascade showing upregulated genes (highlighted in red) in A1 astrocytes derived from POLG patient organoids. Multiple core components of the canonical TGF-β pathway were transcriptionally activated, including ***TGFB1***, ***TGFBR1***, and downstream *SMAD2, SMAD3,* and *SMAD7*. Elevated expression of transcriptional targets such as *FOS, ID1*, and *ZEB2* further supports activation of the TGF-β–SMAD axis. This suggests that *POLG* mutations trigger a reactive astrocyte phenotype through TGF-β–mediated reprogramming, contributing to gliosis, extracellular matrix remodeling, and potential neurotoxic responses. The color scale (top right) reflects log2 fold change values ranging from –2 to +2.

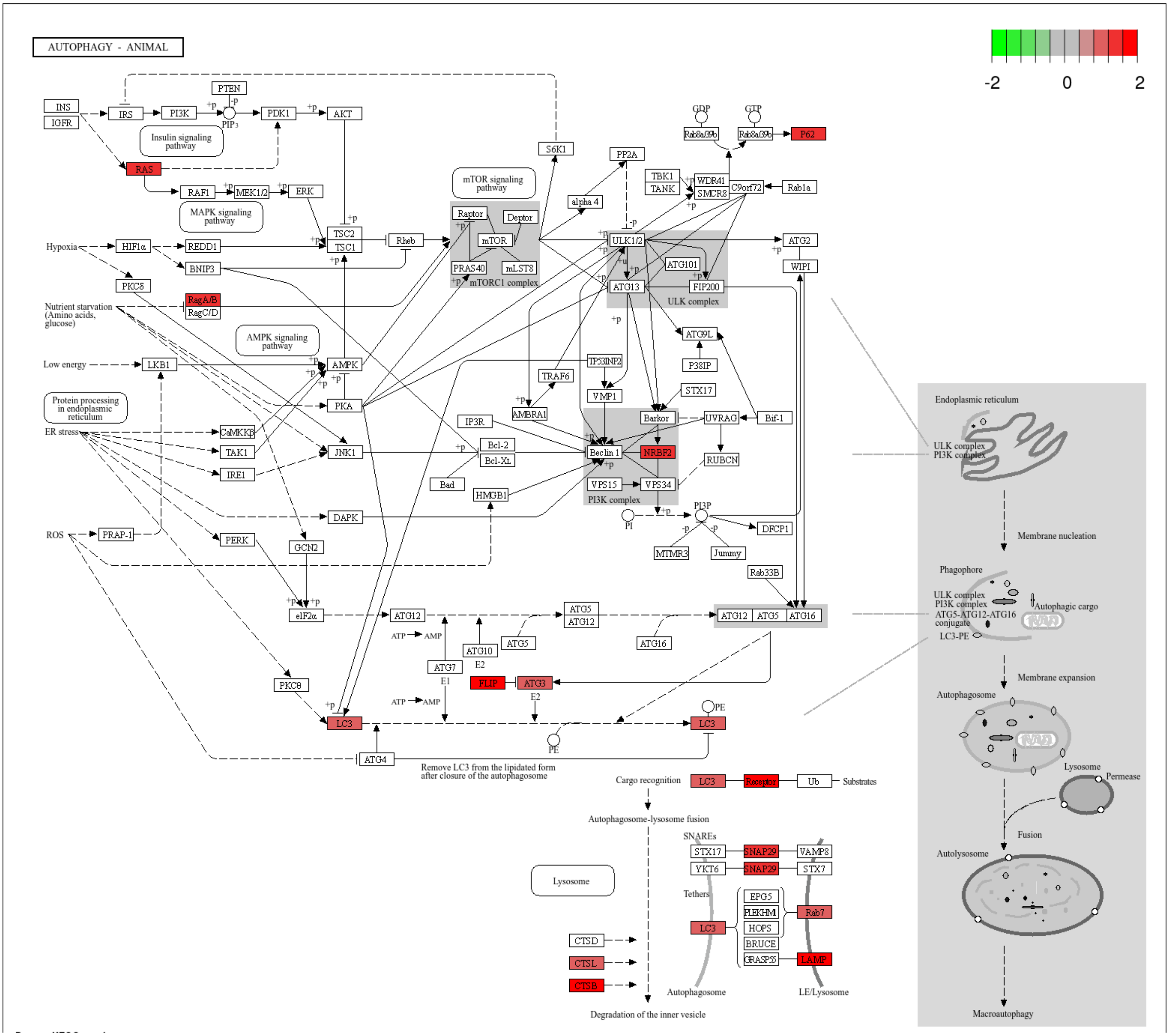

Figure S4. KEGG pathway analysis reveals activation of autophagy-related genes in POLG A1 astrocytes.

Visualization of the KEGG Autophagy – Animal pathway shows upregulation (red) of multiple core components in A1 astrocytes derived from POLG patient organoids. Upregulated genes include *BECN1, ATG3, ATG7, MAP1LC3B (LC3B*), and *GABARAPL1,* which are involved in autophagosome initiation, elongation, and maturation. These findings suggest that POLG astrocytes exhibit increased autophagic activity, likely as an adaptive response to cellular and mitochondrial stress. The pathway diagram also highlights components of lysosomal degradation and membrane trafficking, consistent with enhanced vesicular turnover and protein clearance mechanisms. Color scale (top right) indicates log2 fold changes in gene expression.

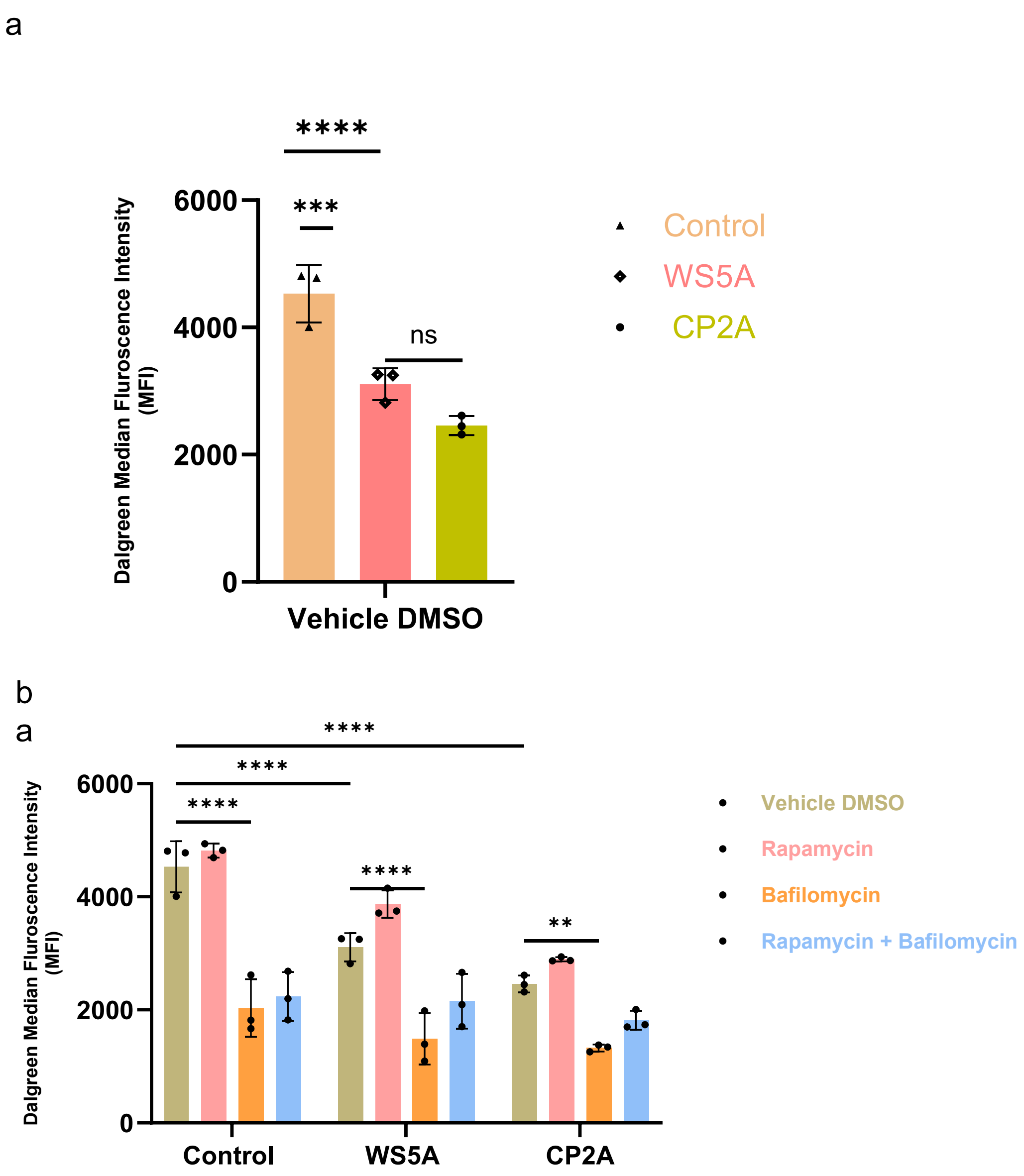

Figure S5. Autophagy activity assessed using a DALGreen-based assay in control and *POLG* mutant astrocytes.

(a) Basal autophagy–lysosome activity measured by DALGreen fluorescence in control and POLG astrocytes (WS5A and CP2A) under vehicle (DMSO) treatment. POLG astrocytes exhibited significantly reduced DALGreen fluorescence compared with control astrocytes, indicating decreased basal autophagy–lysosome activity.
(b) Autophagy modulation assay. Astrocytes were treated with rapamycin (autophagy inducer), bafilomycin A1 (lysosomal inhibitor), or a combination of rapamycin and bafilomycin. DALGreen fluorescence increased following rapamycin treatment, confirming that the autophagy pathway remains responsive to pharmacological induction. In contrast, bafilomycin treatment markedly reduced DALGreen fluorescence due to inhibition of lysosomal acidification required for DALGreen signal generation. Combined rapamycin and bafilomycin treatment suppressed the rapamycin-induced increase in fluorescence. Data are presented as mean ± SEM from independent biological replicates. Statistical significance was determined using the Mann–Whitney U test. $^{**}p < 0.01$, $^{****}p < 0.0001$; ns, not significant.

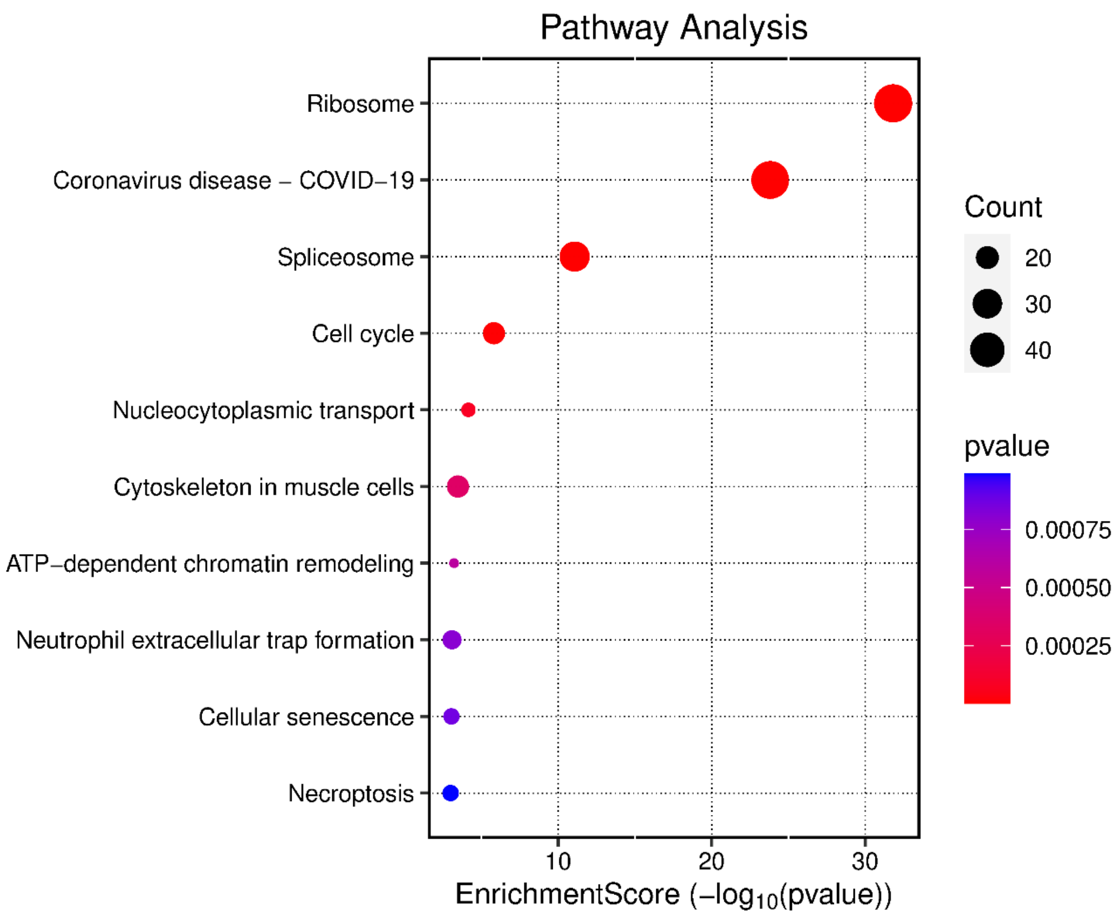

Figure S6. Global KEGG pathway enrichment analysis of upregulated genes in POLG A1 astrocytes.

Dot plot representing significantly enriched KEGG pathways among upregulated genes in POLG patient–derived A1 astrocytes relative to isogenic controls. The top pathways include Ribosome, Coronavirus disease – COVID-19, Spliceosome, and Cell cycle, suggesting increased translational activity, RNA processing, and proliferative stress. Additional enriched pathways—such as nucleocytoplasmic transport, ATP-dependent chromatin remodeling, cellular senescence, necroptosis, and neutrophil extracellular trap formation—indicate heightened transcriptional remodeling, stress signaling, and inflammatory responses. Dot size corresponds to the number of genes enriched in each pathway, while color reflects adjusted p-values (red: most significant).

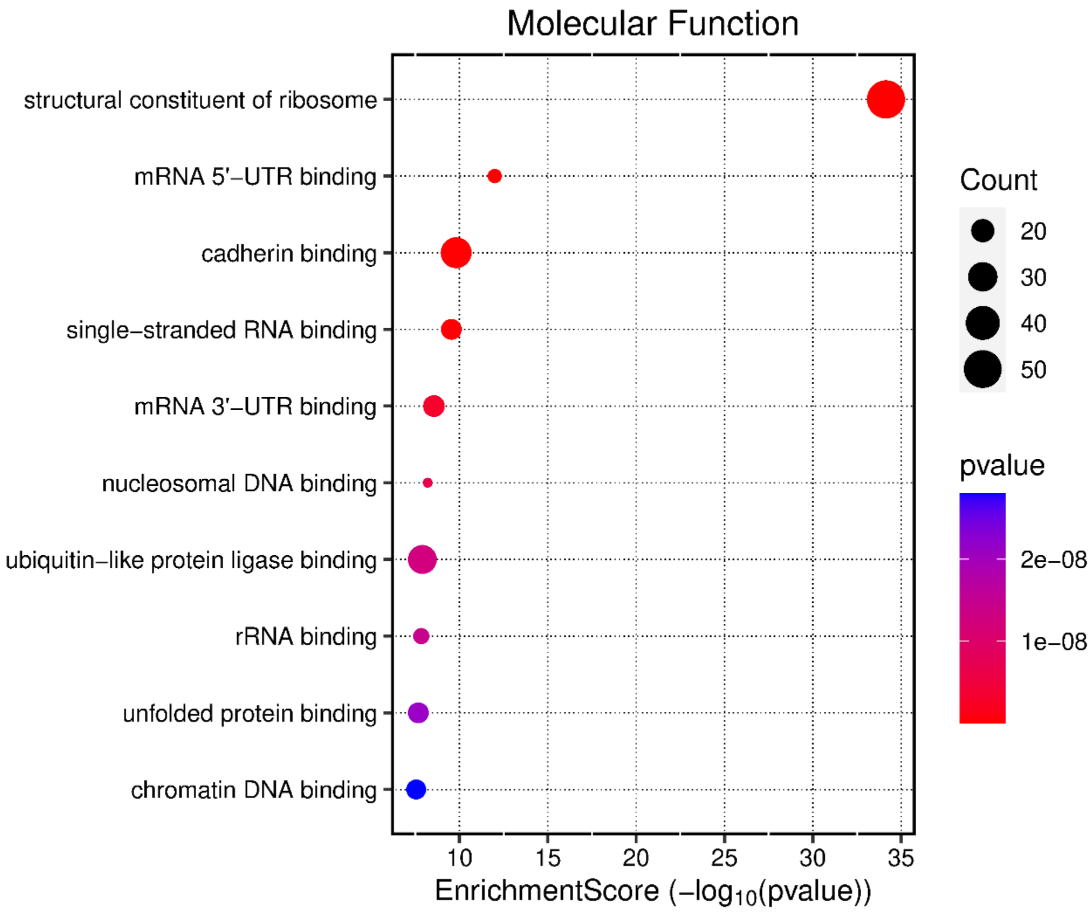

Figure S7. GO Molecular Function enrichment analysis of upregulated genes in POLG A1 astrocytes.

Dot plot depicting significantly enriched molecular functions among genes upregulated in *POLG* mutant A1 astrocytes. The most enriched term, structural constituent of ribosome, indicates increased ribosomal biogenesis or translational stress. Additional highly enriched categories include mRNA 5'-UTR binding, single-stranded RNA binding, and mRNA 3'-UTR binding, suggesting post-transcriptional regulation and stress-adaptive translation. Enrichment of unfolded protein binding, ubiquitin-like protein ligase binding, and chromatin DNA binding points toward disrupted proteostasis and altered epigenetic control. Dot size represents the number of enriched genes, and color gradient indicates statistical significance, with red denoting higher enrichment confidence.

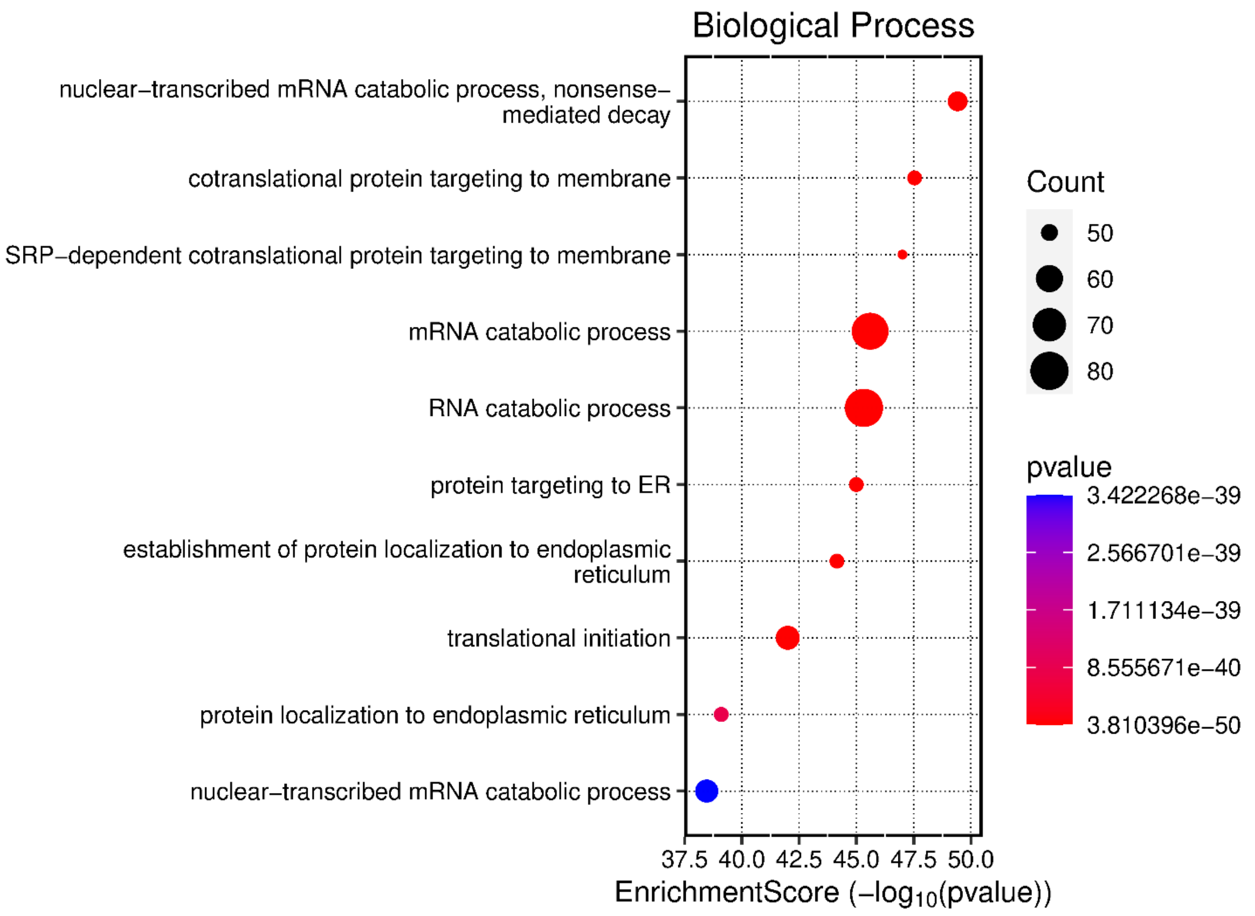

Figure S8. GO Biological Process enrichment analysis of upregulated genes in POLG A1 astrocytes.

Dot plot showing significantly enriched biological processes among upregulated genes in *POLG* mutant A1 astrocytes. The most enriched terms include nuclear-transcribed mRNA catabolic process, nonsense-mediated decay, mRNA catabolic process, and RNA catabolic process, suggesting activation of RNA degradation and surveillance mechanisms. Additional enrichment in cotranslational protein targeting to membrane and translational initiation indicates enhanced protein synthesis and ER targeting, potentially reflecting elevated secretory demands. Dot size corresponds to the number of genes enriched in each category, while color gradient represents statistical significance (red = high confidence).

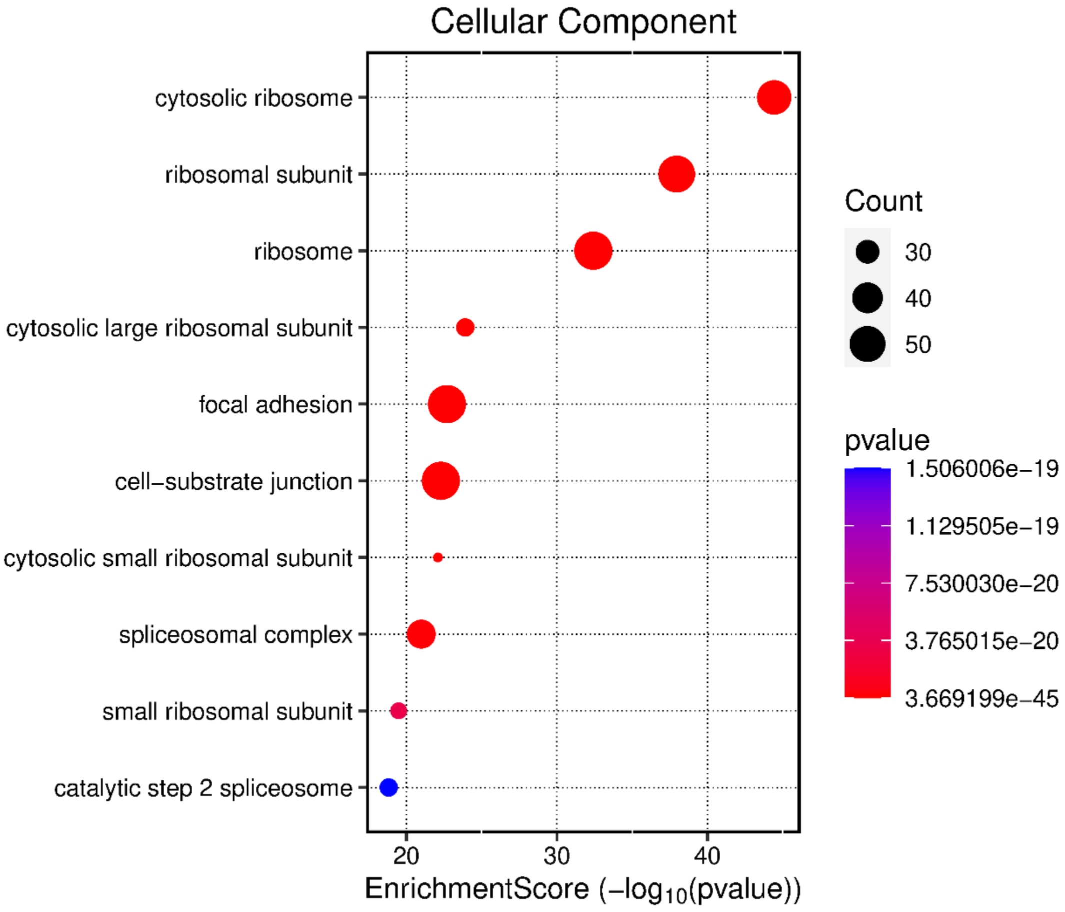

Figure S9. GO Cellular Component enrichment analysis of upregulated genes in POLG A1 astrocytes.

Dot plot showing significantly enriched cellular components among upregulated genes in *POLG* mutant A1 astrocytes. Top enriched terms include cytosolic ribosome, ribosomal subunit, spliceosomal complex, and focal adhesion, indicating increased translational machinery and altered cell–matrix interaction structures. Enrichment in cell-substrate junctions and spliceosomal components suggests remodeling of adhesion and RNA processing compartments under astrocytic stress. Dot size reflects the number of enriched genes per term, and color indicates the statistical significance of enrichment (red = highly significant).

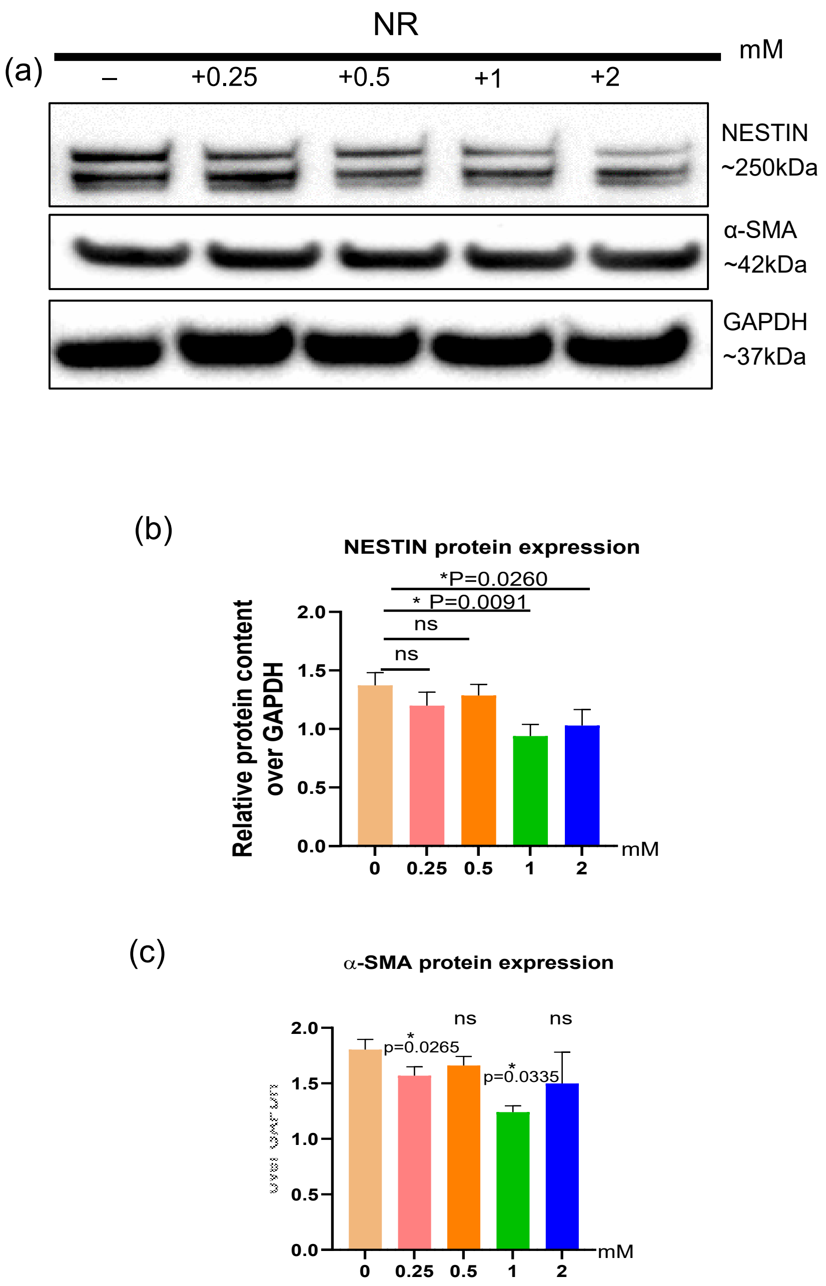

Figure S10. Western blot analysis of NESTIN and α-SMA expression in POLG astrocytes following NR treatment.

(a) Representative Western blot showing NESTIN and α-SMA protein levels in POLG astrocytes treated with increasing concentrations of nicotinamide riboside (NR). GAPDH was used as a loading control. (b–c) Quantification of relative NESTIN and α-SMA protein expression normalized to GAPDH. Statistical significance was determined using the Paired t test. Data are presented as mean ± SEM; *$p < 0.05$; ns, not significant.

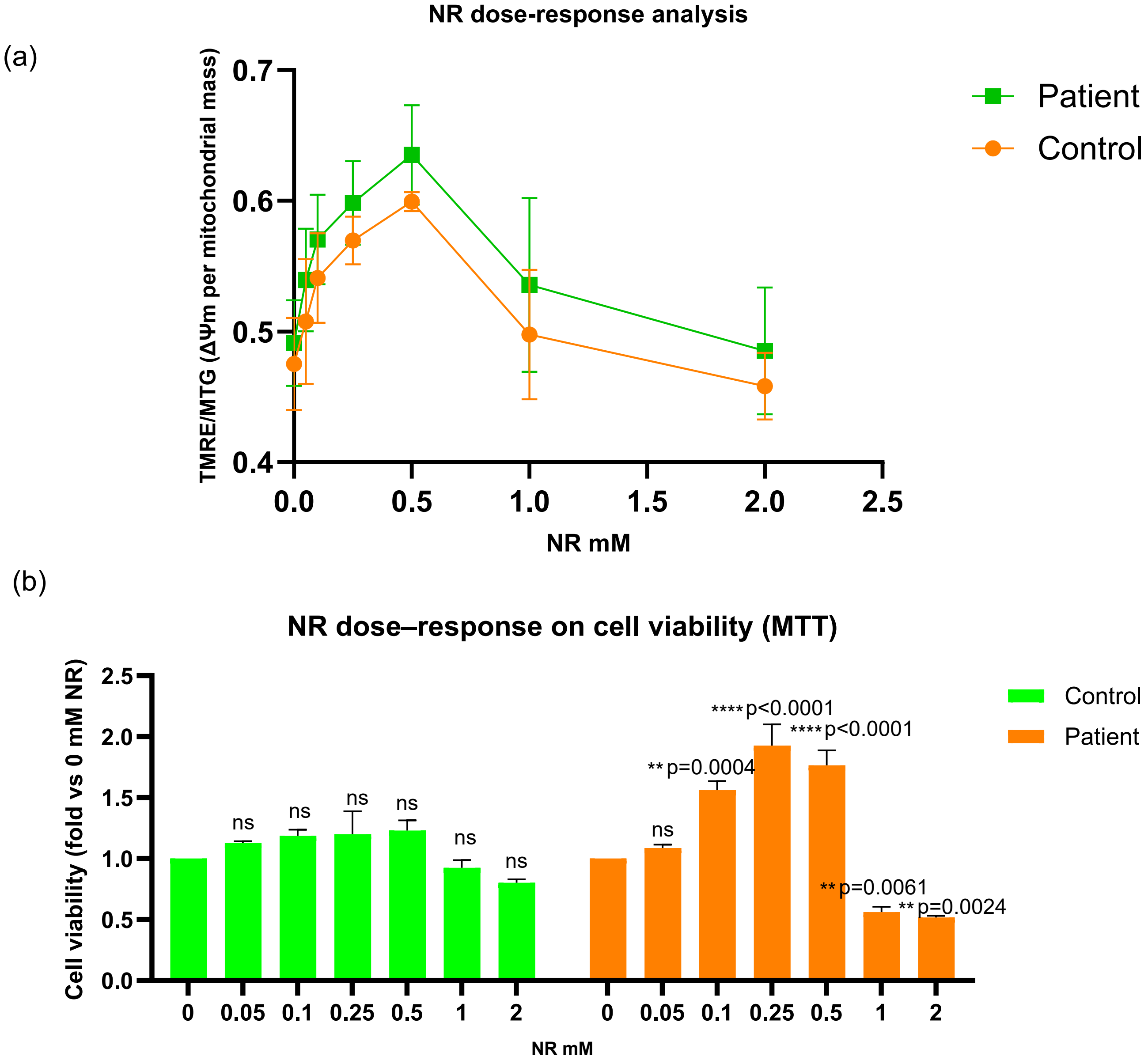

Figure S11. Dose–response effects of NR on mitochondrial function and cell viability.

(a) NR dose–response analysis of mitochondrial membrane potential in control and POLG patient-derived cells. Mitochondrial membrane potential was assessed using TMRE staining and normalized to mitochondrial mass measured by MTG. Cells were treated with increasing concentrations of NR (0–2 mM). Moderate NR concentrations improved mitochondrial membrane potential, with maximal effects observed around 0.5 mM in both control and patient cells, whereas higher concentrations resulted in a decline in mitochondrial function. (b) NR dose–response analysis of cell viability measured by the MTT assay. Cell viability is presented as fold change relative to untreated cells (0 mM NR). Moderate NR concentrations (0.1–0.5 mM) increased cell viability, particularly in patient-derived cells, whereas higher concentrations (1–2 mM) reduced viability. Data are presented as mean ± SEM. Statistical significance was determined using a two-way mixed-effects model (REML) followed by Tukey's multiple comparisons test was used.. **p < 0.01; ****p < 0.000; ns, not significant.

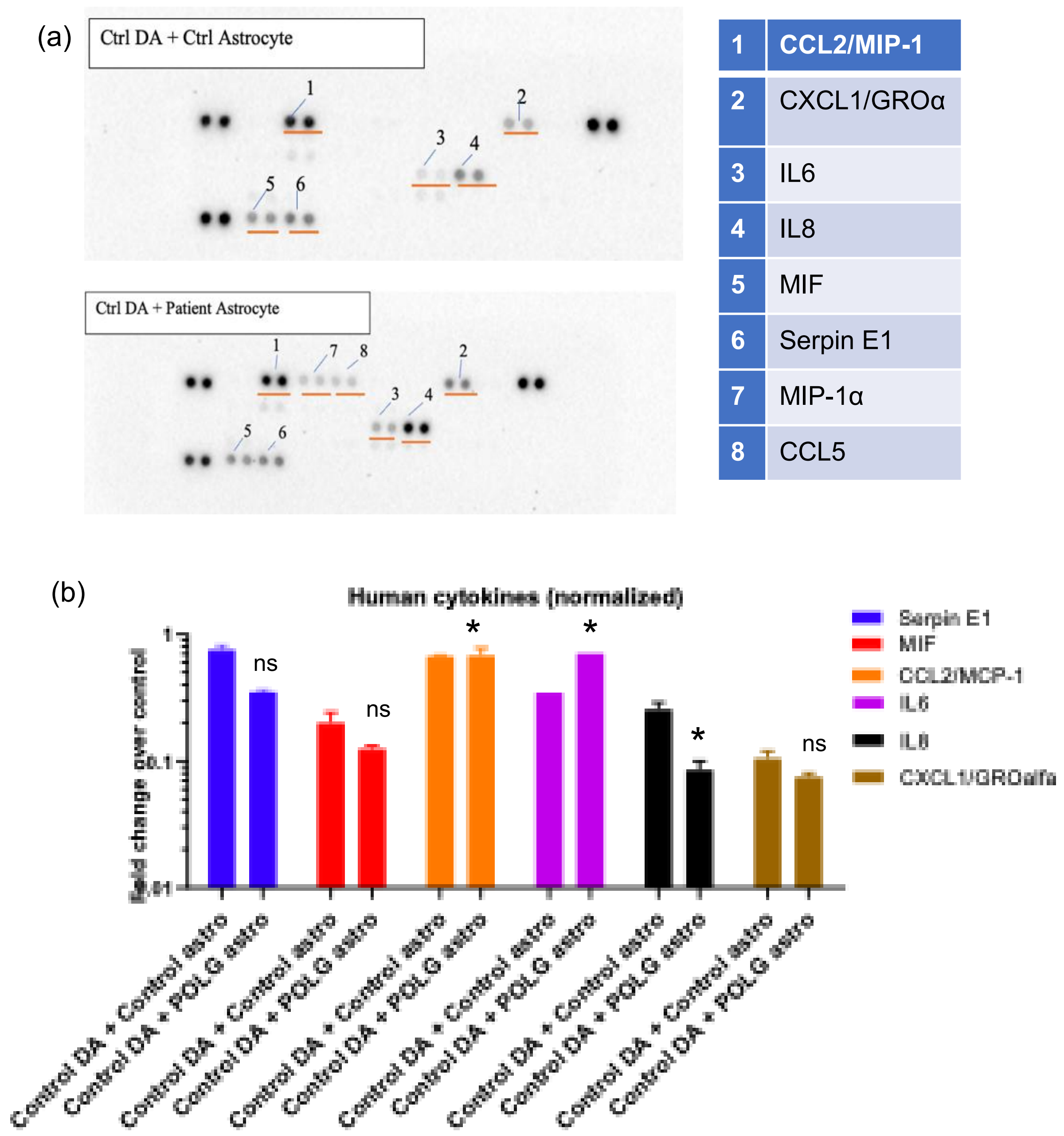

Figure S12. Cytokine profiling of control and POLG-mutant astrocyte–neuron co-cultures.

(a) Representative human cytokine array blots from DA neuron co-cultures with control astrocytes (top) or POLG patient-derived astrocytes (bottom). Numbers indicate cytokines detected, including CCL2/MCP-1, CXCL1/GROα, IL-6, IL-8, MIF, Serpin E1. (b) Quantification of normalized cytokine expression levels across conditions. Co-culture of DA neurons with POLG astrocytes resulted in increased secretion of pro-inflammatory cytokines (e.g., IL-6, CCL2/MCP-1) compared with control astrocytes, consistent with a reactive, neurotoxic astrocytic phenotype. Data are presented as fold change relative to control co-cultures; bars represent mean ± SEM. Statistical significance was determined using the Mann–Whitney U test. *$p < 0.05$; ns, not significant.

(a)

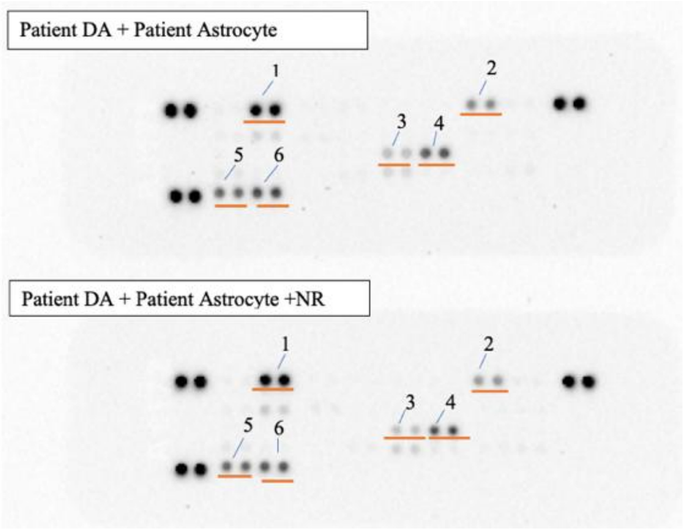

1	**CCL2/MIP-1**
2	CXCL1/GROα
3	IL6
4	IL8
5	MIF
6	Serpin E1

(b)

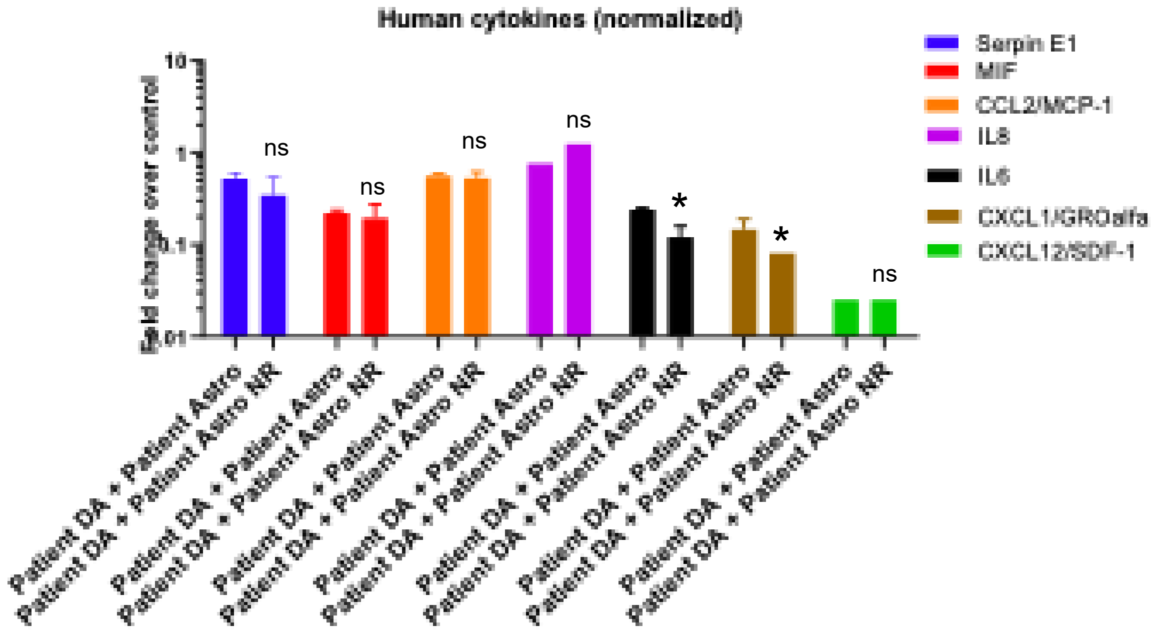

Figure S13. NR reduces pro-inflammatory cytokine secretion in POLG astrocyte–neuron co-cultures.

(a) Representative human cytokine array blots from DA neuron co-cultures with POLG patient-derived astrocytes (top) or with NR treatment (bottom). Detected cytokines include CCL2/MCP-1, CXCL1/GROα, IL-6, IL-8, MIF, and Serpin E1. (b) Quantification of normalized cytokine expression levels across conditions. Co-culture of DA neurons with POLG astrocytes resulted in elevated secretion of inflammatory cytokines (IL-8), consistent with astrocyte reactivity. NR treatment significantly reduced cytokine release IL-6 and CXCL1/GROα, indicating a protective effect against astrocyte-mediated neuroinflammatory signaling. Data are presented as fold change relative to control; bars represent mean ± SEM. Statistical significance was determined using the Mann–Whitney U test. *$p < 0.05$; ns, not significant.

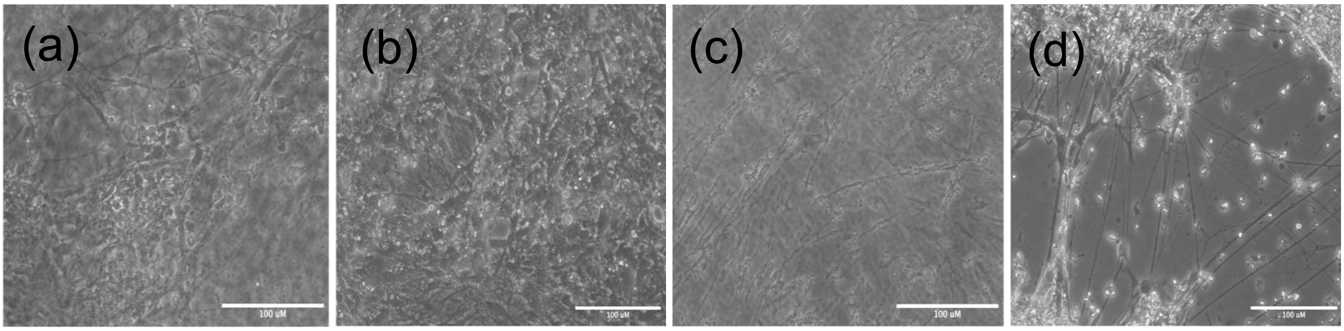

Figure S14. Representative brightfield images of dopaminergic neurons co-cultured with astrocytes at day 10.

Brightfield microscopy images show the morphology of DA neurons cultured under different astrocyte co-culture conditions. From left to right: (a) Control DA neurons with control astrocytes, exhibiting dense neurite networks and healthy somatic structure; (b) Control DA neurons with POLG astrocytes, showing disrupted morphology, reduced neurite complexity, and signs of degeneration; (c) POLG DA neurons with control astrocytes, exhibiting moderate neurite outgrowth; (d) POLG DA neurons with POLG astrocytes, displaying sparse, retracted processes and unhealthy morphology. Scale bars: 100 µm. These images highlight the differential impact of astrocyte genotype on neuronal integrity.

Table S1. Detailed list of A1-associated genes corresponding to Fig. 3D.

names	logfoldchanges	pvals	pvals_adj	pct_nz_group	pct_nz_reference
CD63	1.567432	0	0	0.995666	0.967219
SH3BGRL3	1.884409	0	0	0.992198	0.926548
ANXA2	1.947167	0	0	0.999711	0.994233
CTSB	2.628202	0	0	0.9771	0.735387
LGALS1	2.809057	0	0	0.99935	0.847926
CCN2	3.09118	0	0	0.997761	0.830056
GAP43	3.956804	0	0	0.959185	0.384921
PLAT	4.038429	0	0	0.936936	0.290372
SERPINE1	4.738181	0	0	0.98548	0.360596
CXCL8	5.749462	0	0	0.935997	0.12237

Table S2. Detailed list of KEGG pathway enrichment in A1-associated genes correspo nding to Fig. 3E.

ID	Description	logfold changes	P-value	Adjusted P-value	Odds Ratio	Combined Score
GO_Biological_Process_2021	cytokine-mediated signaling pathway (GO:0019221)	27.19003308	8.86646E-31	6.45605E-28	3.797829515	262.8016831
GO_Biological_Process_2021	regulation of apoptotic process (GO:0042981)	22.35688908	1.12135E-25	4.39654E-23	3.17990755	182.685999
GO_Biological_Process_2021	cellular response to cytokine stimulus (GO:0071345)	17.01515916	5.11552E-20	9.65697E-18	3.407731627	151.3694705
GO_Biological_Process_2021	Ras protein signal transduction (GO:0007265)	16.71216467	1.06581E-19	1.94015E-17	6.829095984	298.3316814
GO_Biological_Process_2021	regulation of intracellular signal transduction (GO:1902531)	16.43639617	2.08299E-19	3.66103E-17	3.496154074	150.3881596
GO_Biological_Process_2021	regulation of I-kappaB kinase/NF-kappaB signaling (GO:0043122)	16.18553024	3.9675E-19	6.52334E-17	4.942198992	209.4058188
GO_Biological_Process_2021	regulation of MAPK cascade (GO:0043408)	12.21241276	6.1354E-15	6.13179E-13	4.97881664	162.9302876
GO_Biological_Process_2021	regulation of autophagy (GO:0010506)	11.84544687	1.51228E-14	1.42742E-12	4.087413521	130.0720141
GO_Biological_Process_2021	positive regulation of apoptotic process (GO:0043065)	10.33642335	6.32936E-13	4.60868E-11	3.299047033	92.66497409
GO_Biological_Process_2021	regulation of inflammatory response (GO:0050727)	10.27988011	7.46513E-13	5.24952E-11	4.029809316	112.5258295
GO_Biological_Process_2021	ERAD pathway (GO:0036503)	8.693189725	4.13551E-11	2.0268E-09	6.039115578	144.388161
GO_Biological_Process_2021	cellular response to hypoxia (GO:0071456)	7.96301902	2.75585E-10	1.08888E-08	4.486283989	98.75263901
GO_Biological_Process_2021	negative regulation of Wnt signaling pathway (GO:0030178)	7.954418091	2.83277E-10	1.11066E-08	3.64513646	80.13685784
GO_Biological_Process_2021	response to endoplasmic reticulum stress (GO:0034976)	7.84158596	3.81446E-10	1.44017E-08	4.898989899	106.2446466
GO_Biological_Process_2021	regulation of transcription. DNA-templated (GO:0006355)	7.83632646	3.91814E-10	1.45772E-08	1.618255556	35.05179559
GO_Biological_Process_2021	regulation of neuron projection development (GO:0010975)	6.54328703	1.10628E-08	2.86229E-07	3.545038634	64.9439693
GO_Biological_Process_2021	response to tumor necrosis factor (GO:0034612)	6.136431838	3.18131E-08	7.30412E-07	4.263263024	73.59835734
GO_Biological_Process_2021	cellular response to oxidative stress (GO:0034599)	5.55548984	1.43599E-07	2.78298E-06	3.755060662	59.16564251
GO_Biological_Process_2021	regulation of actin cytoskeleton organization (GO:0032956)	5.238406199	3.39939E-07	5.77556E-06	4.302500308	64.08359497

Table S3. Detailed list of neural related GO/BP downregulated in A1 astrocytes in patient vs co ntrol corresponding to Fig. 4B.

ID	Description	logfold changes	GeneRatio	BgRatio	pvalue	p.adjust	qvalue
GO:0048713	regulation of oligodendrocyte differentiation	3.710924301	8/498	40/18866	8.15E-06	0.000234	0.000195
GO:2000179	positive regulation of neural precursor cell proliferation	2.189894017	7/498	54/18866	0.000518	0.00776	0.006458
GO:0099188	postsynaptic cytoskeleton organization	2.189894017	4/498	15/18866	0.000519	0.00776	0.006458
GO:0021915	neural tube development	1.850118554	12/498	163/18866	0.001329	0.016967	0.014122
GO:0001841	neural tube formation	1.712522965	9/498	106/18866	0.001975	0.023292	0.019386
GO:2000177	regulation of neural precursor cell proliferation	1.595942943	8/498	91/18866	0.002789	0.030464	0.025355
GO:0098974	postsynaptic actin cytoskeleton organization	1.449699003	3/498	13/18866	0.004292	0.042661	0.035506
GO:0050769	positive regulation of neurogenesis	1.38444602	23/498	485/18866	0.005166	0.049578	0.041262
GO:0050768	negative regulation of neurogenesis	1.116123897	15/498	295/18866	0.012115	0.091962	0.076538
GO:0097150	neuronal stem cell population maintenance	0.931418595	3/498	23/18866	0.021871	0.140707	0.117107
GO:0051402	neuron apoptotic process	0.836748919	12/498	245/18866	0.029963	0.174979	0.14563
GO:0043696	dedifferentiation	0.768048314	2/498	12/18866	0.038527	0.204968	0.170589
GO:0043697	cell dedifferentiation	0.768048314	2/498	12/18866	0.038527	0.204968	0.170589
GO:0014009	glial cell proliferation	0.738413128	4/498	50/18866	0.042573	0.219443	0.182636
GO:0045666	positive regulation of neuron differentiation	0.726408971	16/498	380/18866	0.045757	0.225593	0.187755

Table S4. Detailed list of neural related GO/CC downregulated in A1 astrocyte in patient vs control corresponding to Fig. 4C.

ID	description	logfold changes	generatio	bgratio	pvalue	p.adjust	qvalue
GO:0005874	microtubule	3.585236746	27/512	431/19559	2.89636E-05	0.000348943	0.000259874
GO:0014069	postsynaptic density	1.828335069	18/512	337/19559	0.003546076	0.019936826	0.014847896
GO:0032432	actin filament bundle	1.811716363	7/512	76/19559	0.003725337	0.020714513	0.015427077
GO:0098984	neuron to neuron synapse	1.783761093	19/512	368/19559	0.004016681	0.022091744	0.016452765

Table S5. Detailed list of neural related GO/BP downregulated in A1 astrocytes in patient vs control corresponding to Fig. 4D (part 1).

ID	*description*	logfold changes	*generatio*	*bgratio*	*pvalue*	*p.adjust*	*qvalue*
GO:0072655	establishment of protein localization to mitochondrion	5.176233386	17/498	140/18866	1.75E-07	8.01E-06	6.66E-06
GO:0070585	protein localization to mitochondrion	5.03537644	17/498	144/18866	2.64E-07	1.11E-05	9.22E-06
GO:0043044	ATP-dependent chromatin remodeling	3.939055322	12/498	90/18866	4.24E-06	0.000138	0.000115
GO:0007006	mitochondrial membrane organization	3.850197047	15/498	142/18866	5.4E-06	0.00017	0.000141
GO:0006626	protein targeting to mitochondrion	3.573768509	12/498	99/18866	1.15E-05	0.000321	0.000267
GO:0051204	protein insertion into mitochondrial membrane	3.37303323	8/498	45/18866	2.03E-05	0.000509	0.000424
GO:0006839	mitochondrial transport	3.150587643	20/498	271/18866	3.71E-05	0.000849	0.000707
GO:0090151	establishment of protein localization to mitochondrial membrane	3.13582194	8/498	49/18866	3.88E-05	0.000879	0.000731
GO:0006979	response to oxidative stress	2.801858404	27/498	458/18866	9.3E-05	0.001896	0.001578
GO:1901028	regulation of mitochondrial outer membrane permeabilization involved in apoptotic signaling pathway	2.586115295	7/498	45/18866	0.000163	0.003116	0.002593
GO:0008637	apoptotic mitochondrial changes	2.214405196	11/498	125/18866	0.000481	0.007334	0.006104
GO:0097345	mitochondrial outer membrane permeabilization	2.150341452	7/498	55/18866	0.000581	0.008499	0.007074
GO:1903747	regulation of establishment of protein localization to mitochondrion	2.093968901	8/498	73/18866	0.000666	0.009678	0.008054
GO:1903749	positive regulation of establishment of protein localization to mitochondrion	2.026444579	7/498	58/18866	0.000804	0.011305	0.009409
GO:1902110	positive regulation of mitochondrial membrane permeability involved in apoptotic process	1.914918567	7/498	61/18866	0.00109	0.014616	0.012164

Table S6. Detailed list of Neural related GO/BP downregulated in A1 astrocytes in patient vs control corresponding to Fig. 4D (part 2).

ID	*description*	logfold changes	*generatio*	*bgratio*	*pvalue*	*p.adjust*	*qvalue*
GO:1902686	mitochondrial outer membrane permeabilization involved in programmed cell death	1.850118554	7/498	63/18866	0.001321	0.016967	0.014122
GO:0030150	protein import into mitochondrial matrix	1.842399215	4/498	19/18866	0.001356	0.017272	0.014375
GO:0035794	positive regulation of mitochondrial membrane permeability	1.782559157	7/498	65/18866	0.001589	0.019823	0.016498
GO:1902108	regulation of mitochondrial membrane permeability involved in apoptotic process	1.751145269	7/498	66/18866	0.001738	0.02131	0.017736
GO:0034614	cellular response to reactive oxygen species	1.722280662	12/498	170/18866	0.001899	0.022775	0.018955
GO:1901030	positive regulation of mitochondrial outer membrane permeabilization involved in apoptotic signaling pathway	1.691276103	5/498	35/18866	0.00212	0.02446	0.020357
GO:0007007	inner mitochondrial membrane organization	1.649681035	6/498	52/18866	0.002389	0.026919	0.022404
GO:0046902	regulation of mitochondrial membrane permeability	1.451968108	7/498	77/18866	0.004191	0.042439	0.035321
GO:1900739	regulation of protein insertion into mitochondrial membrane involved in apoptotic signaling pathway	1.433026101	4/498	26/18866	0.004522	0.044331	0.036896
GO:1900740	positive regulation of protein insertion into mitochondrial membrane involved in apoptotic signaling pathway	1.433026101	4/498	26/18866	0.004522	0.044331	0.036896
GO:0001844	protein insertion into mitochondrial membrane involved in apoptotic signaling pathway	1.263211837	4/498	30/18866	0.007629	0.065542	0.054549
GO:0010822	positive regulation of mitochondrion organization	1.084598366	8/498	119/18866	0.013647	0.098886	0.0823
GO:0010821	regulation of mitochondrion organization	0.833525337	10/498	191/18866	0.030677	0.176282	0.146715
GO:0015867	ATP transport	0.776847322	3/498	28/18866	0.03674	0.200857	0.167168
GO:1903580	positive regulation of ATP metabolic process	0.738413128	4/498	50/18866	0.042573	0.219443	0.182636

Table S7. Detailed list of mitochondrial related GO/CC downregulated in A1 astrocyte in patient vs control corresponding to Fig. 4E.

ID	description	logfold changes	generatio	bgratio	pvalue	p.adjust	qvalue
GO:0098798	mitochondrial protein complex	4.057248055	21/512	265/19559	7.21031E-06	0.000117691	8.765E-05
GO:0005743	mitochondrial inner membrane	2.789883882	27/512	489/19559	0.000237344	0.002178247	0.001622244
GO:0098799	outer mitochondrial membrane protein complex	2.6714852	5/512	24/19559	0.00033924	0.002860921	0.002130663
GO:0032592	integral component of mitochondrial membrane	2.175094483	8/512	81/19559	0.001258938	0.008972152	0.006681985
GO:0098573	intrinsic component of mitochondrial membrane	2.146435686	8/512	82/19559	0.001363758	0.00958419	0.007137799
GO:0005758	mitochondrial intermembrane space	1.720243453	7/512	80/19559	0.004952485	0.025570996	0.019043929
GO:0005744	TIM23 mitochondrial import inner membrane translocase complex	1.709330425	3/512	14/19559	0.005233974	0.02622169	0.019528531
GO:0031304	intrinsic component of mitochondrial inner membrane	1.501794241	5/512	50/19559	0.009694029	0.042286024	0.0314924
GO:0031305	integral component of mitochondrial inner membrane	1.501794241	5/512	50/19559	0.009694029	0.042286024	0.0314924
GO:0005741	mitochondrial outer membrane	1.07162567	10/512	192/19559	0.030152249	0.113858492	0.084795798
GO:0005759	mitochondrial matrix	0.943349843	19/512	473/19559	0.043823351	0.152982324	0.113933164

Table S8. Detailed list of KEGG pathways upregulated in A1 astrocyte in patient vs control corresponding to Fig. 5A.

ID	description	logfold changes	generatio	bgratio	pvalue	p.adjust	qvalue
hsa04218	Cellular senescence	1.621441818	12/234	157/8846	0.000912724	0.028292106	0.023908822
hsa04350	TGF-beta signaling pathway	1.093216971	8/234	108/8846	0.007732138	0.095475101	0.080683184
hsa04140	Autophagy - animal	0.959834978	10/234	169/8846	0.014168226	0.129799231	0.109689491
hsa04216	Ferroptosis	0.824490723	4/234	42/8846	0.024342358	0.177262298	0.149799125
hsa04010	MAPK signaling pathway	0.784635795	14/234	300/8846	0.028050255	0.19429933	0.164196617
hsa04724	Glutamatergic synapse	0.744180333	7/234	116/8846	0.033969432	0.213268525	0.180226923
hsa04550	Signaling pathways regulating pluripotency of stem cells	0.740576386	8/234	144/8846	0.037102951	0.215045675	0.18172874

Table S9. Detailed list of immune related GO/BP downregulated in A1 astrocyte in patient vs control corresponding to Fig. 5B.

ID	description	logfold changes	generatio	bgratio	pvalue	p.adjust	qvalue
GO:0002367	cytokine production involved in immune response	1.751145	9/498	104/18866	0.001731	0.02131	0.017736
GO:0002720	positive regulation of cytokine production involved in immune response	1.556068	6/498	55/18866	0.003184	0.033394	0.027793
GO:0061640	cytoskeleton-dependent cytokinesis	1.399268	8/498	100/18866	0.004976	0.047914	0.039878
GO:0002718	regulation of cytokine production involved in immune response	1.324192	7/498	83/18866	0.006326	0.056956	0.047403
GO:0000910	cytokinesis	1.030002	10/498	172/18866	0.016169	0.112133	0.093325
GO:0032506	cytokinetic process	0.977501	4/498	39/18866	0.019025	0.126541	0.105317
GO:0050856	regulation of T cell receptor signaling pathway	0.872816	4/498	43/18866	0.026303	0.161034	0.134025
GO:0042104	positive regulation of activated T cell proliferation	0.83576	3/498	26/18866	0.030302	0.175378	0.145962
GO:0002730	regulation of dendritic cell cytokine production	0.768048	2/498	12/18866	0.038527	0.204968	0.170589
GO:0035739	CD4-positive. alpha-beta T cell proliferation	0.768048	2/498	12/18866	0.038527	0.204968	0.170589
GO:2000561	regulation of CD4-positive. alpha-beta T cell proliferation	0.768048	2/498	12/18866	0.038527	0.204968	0.170589
GO:0061082	myeloid leukocyte cytokine production	0.73168	3/498	30/18866	0.043819	0.222871	0.18549
GO:0002371	dendritic cell cytokine production	0.73168	2/498	13/18866	0.044751	0.222871	0.18549

Table S9. Detailed list of GO/BP upregulated in A1 astrocyte in patient vs control corresponding to Fig. 5B.

ID	description	logfold changes	generatio	bgratio	pvalue	p.adjust	qvalue
GO:0043312	neutrophil degranulation	6.354102074	35/394	487/18866	2.16622E-10	5.4859E-07	4.42484E-07
GO:0002283	neutrophil activation involved in immune response	6.354102074	35/394	490/18866	2.55694E-10	5.4859E-07	4.42484E-07
GO:0035722	interleukin-12-mediated signaling pathway	1.439156865	5/394	47/18866	0.002890464	0.045101755	0.036378362
GO:0071349	cellular response to interleukin-12	1.39861367	5/394	49/18866	0.003473319	0.049514984	0.039938002
GO:0070671	response to interleukin-12	1.377197483	5/394	50/18866	0.003794361	0.052017902	0.041956815
GO:0032651	regulation of interleukin-1 beta production	1.008213045	6/394	97/18866	0.016075401	0.121657046	0.098126646
GO:0001865	NK T cell differentiation	1.002831651	2/394	10/18866	0.017522294	0.123173891	0.099350109
GO:0002664	regulation of T cell tolerance induction	1.002831651	2/394	10/18866	0.017522294	0.123173891	0.099350109
GO:0001916	positive regulation of T cell mediated cytotoxicity	0.961360264	3/394	28/18866	0.020117359	0.135515835	0.109304926
GO:0070106	interleukin-27-mediated signaling pathway	0.95753813	2/394	11/18866	0.021123564	0.136713745	0.110271141
GO:0070757	interleukin-35-mediated signaling pathway	0.95753813	2/394	11/18866	0.021123564	0.136713745	0.110271141
GO:0032611	interleukin-1 beta production	0.923880442	6/394	106/18866	0.023789724	0.147730398	0.119156999
GO:0032731	positive regulation of interleukin-1 beta production	0.922545138	4/394	53/18866	0.024571974	0.148185317	0.119523929
GO:0002517	T cell tolerance induction	0.922545138	2/394	12/18866	0.025002603	0.148185317	0.119523929
GO:0002526	acute inflammatory response	0.888859405	6/394	111/18866	0.028995106	0.160136712	0.129163735
GO:0032652	regulation of interleukin-1 production	0.888859405	6/394	111/18866	0.028995106	0.160136712	0.129163735
GO:0072683	T cell extravasation	0.888859405	2/394	13/18866	0.0291463	0.160136712	0.129163735
GO:0001914	regulation of T cell mediated cytotoxicity	0.836991475	3/394	35/18866	0.036135401	0.18045081	0.145548765
GO:0032732	positive regulation of interleukin-1 production	0.834152239	4/394	60/18866	0.036572386	0.181634387	0.146503419
GO:0032612	interleukin-1 production	0.805303648	6/394	121/18866	0.041527026	0.194109443	0.156565602
GO:0002716	negative regulation of natural killer cell mediated immunity	0.774977527	2/394	17/18866	0.048120036	0.20814826	0.167889089

Table S10. Detailed list of immune response related genes corresponding to Fig. 5C.

names	logfold changes	pvals	pvals_adj	pct_nz_group	scores
CXCL8	7.803859	3.88E-46	4.85E-42	0.966475	14.26002
IFITM2	3.52765	2.64E-27	2.54E-24	0.825581	10.82429
HLA-C	3.152368	1.09E-29	1.27E-26	0.88659	11.31646
CCL2	2.847795	2.35E-06	0.000164	0.443295	4.72089
HLA-E	2.619551	1.95E-08	2.05E-06	0.490109	5.616421
HLA-B	2.459084	1.18E-24	8.32E-22	0.921247	10.25055
IFITM3	1.658153	3.64E-25	2.78E-22	0.972969	10.36341
NQO1	1.103557	8.46E-09	9.36E-07	0.885609	5.759021
